# Perinatal interventions to prevent Adverse Childhood Experiences (ACEs): A scoping review

**DOI:** 10.1371/journal.pone.0307441

**Published:** 2024-10-24

**Authors:** Jane Kinsey, Jaime La Charite, Shirley Russ, Adam Schickedanz

**Affiliations:** 1 Center for Healthier Children, Families and Communities, University of California, Los Angeles (UCLA), Los Angeles, CA, United States of America; 2 Department of Pediatrics, Geffen School of Medicine, UCLA, Los Angeles, CA, United States of America; 3 Senior Harkness Fellow, The Commonwealth Fund, New York, NY, United States of America; 4 Department of General Internal Medicine, University of California, Los Angeles, Los Angeles, CA, United States of America; Caribbean Center for Child Neurodevelopment, GRENADA

## Abstract

**Background:**

Preventing Adverse Childhood Experiences (ACEs) is a public health priority, and the perinatal period is a sensitive life stage when preventive interventions could be particularly effective. Protecting and buffering pregnant persons and infants from exposure to adversity can optimize children’s development and health trajectories, reduce future morbidity and mortality, and even break intergenerational cycles of adversity, but no study has synthesized experimental evidence on effectiveness of interventions to address ACEs in the perinatal period.

**Objectives:**

To (1) identify perinatal ACE prevention interventions, tested in high quality randomized control trials, with a dyadic perspective examining outcomes for mother and child; (2) describe their (a) place on the public health prevention continuum and (b) incorporation of life course characteristics that aim to optimize life health trajectories; and (3) determine which interventions show evidence of effectiveness.

**Methods:**

We undertook a scoping review, using a modified PRISMA-Sc approach, of articles published in English between January 2000 and November 2023 identified through Psych info and PubMed using search terms for a broad range of adversities, with additional capture of articles from relevant reference lists. Interventions were included if they targeted an identified ACEs exposure or risk; were tested in randomized controlled trials (RCTs); reported outcome measures for both mother and child and were initiated during pregnancy. Interventions were further analyzed using the public health prevention continuum and Life Course Intervention Research (LCIR) characteristics frameworks. A two-tailed t test was used to ascertain the association between LCIR characteristics, and the outcomes achieved.

**Results:**

Of 2148 articles identified, 57 were in scope for detailed analysis, yielding 53 unique interventions. Overall, 42 (74%) reported some positive impact; 37 (65%) for mothers; 37 (65%) for the child, and 32 (56%) for both. Interventions with the strongest evidence based on study quality and reported outcomes were co-parenting programs designed to improve the quality and function of the co-parenting relationship, home visiting interventions, and integrative health interventions incorporating baby massage and/or yoga. Half of effective interventions were secondary prevention focused. The mean number of life course characteristics was significantly higher in the studies that reported a positive impact on the mother and/or child (p = 0.003).

**Conclusions:**

Few studies specifically addressed ACEs as a defined set of adversities, yet a range of perinatal interventions showed positive impacts on individual ACE risks or exposures. Intentional incorporation of life course characteristics and bundling of evidence-based components into comprehensive perinatal interventions hold promise for future ACEs prevention.

## Introduction

Adverse Childhood Experiences (ACEs) have a clear association with a wide range of adult health conditions and risk-taking behaviors, showing a graded dose-response relationship [1]. Advances in neurobiology, epigenetics, and life course theory have resulted in the recognition of plausible mechanisms whereby adversities experienced early in life can impact a child’s developing biological, psychological, and social processes [2, 3]. Once established in early life, these processes may be difficult to alter, especially if problems go unrecognized for many years [3].

ACEs are common, with one recent estimate suggesting that 61% of US adults experience at least one ACE, and almost 1 in 6 experience 4 or more [4]. The total annual costs attributable to ACEs have been estimated at $581 billion in Europe (equivalent to 2·7% of GDP) and $748 billion (3·6% of GDP) in North America [5]. Acting early to prevent and mitigate ACEs could improve population health across the whole of the life course, prevent considerable suffering and significantly reduce costs, making this a public health priority [4]. Yet many questions remain for both policymakers and providers about the best ways to address ACEs at population level, with prevention being especially challenging [6, 7].

### Public health prevention continuum

The public health prevention continuum provides a useful framework for researchers and interventionists considering how to best direct their efforts to prevent or mitigate the impact of ACEs on lifelong health [8]. Primary prevention efforts are designed to either prevent or reduce the likelihood of ACEs occurring in the first place. These efforts acknowledge that ACEs can develop due to structural and Social Determinants of Health (SDOH) factors such as historic, local, and political contexts where there are multiple stressors on families–racism, discrimination, lack of affordable housing and barriers to childcare–and can include efforts to act on these ‘upstream’ factors [9–15]. Secondary prevention initiatives have an early detection focus, where, after ACEs have occurred but prior to the onset of identifiable symptoms, those who have been exposed to ACEs are identified and supported to access interventions designed to reduce the risk of any sequelae [16, 17]. Tertiary prevention focuses on treatment for families, children and young people who are identified as having already experienced ACEs *and* their impacts, with the goal to mitigate worsening consequences [18]. While all three levels of preventive interventions have a place in a robust multi-pronged approach that can truly impact the prevalence and downstream effects of ACEs, understanding which interventions have evidence of effectiveness on each level of the prevention continuum will help determine which interventions to apply at each level, and which levels of prevention should be an intervention priority. In addition, life course frameworks suggest other useful considerations for the design of effective ACEs prevention interventions.

## Life course health development approach

The Life Course Health Development (LCHD) approach, incorporates the Socio Ecological Model, and suggests that a person’s health results from a series of complex, adaptive relational interactions between their biology, their behaviors, and social and environmental conditions [3, 19, 20]. This means that Interventions designed to improve health trajectories must consider each person’s individual traits, their family and community environment, and the importance of relationships between and across these layers [21]. The approach acknowledges the importance of social and structural determinants of health in the genesis of ACEs, and in the persistence of intergenerational cycles of adversity. For example, early life adversity and toxic stress can have a negative impact on maternal physical and mental health, making it more difficult for a mother to provide nurturing care to her infant and prevent further adverse experiences. Recent work to apply life course principles to intervention research has resulted in the proposal of 12 characteristics of life course interventions and a Life Course Intervention Research (LCIR) Framework yet this framework has yet to be applied to an analysis of ACEs preventive interventions. Understanding whether these life course characteristics are associated with more effective interventions will help to determine the utility of this approach, and to further refine these characteristics as evidence accrues [22].

### Perinatal interventions

Although preconception is a logical stage of the life course to consider ACEs prevention for the next generation, inconsistent contact with the health system and lack of an obvious delivery platform, make interventions in that phase of life challenging [23]. Instead, researchers interested in ACEs prevention have often focused on pregnancy and the perinatal period as the earliest time when expectant parents have frequent contact with the health care system, are engaged with efforts to achieve the best outcomes for their child, and may be most adaptable and motivated to change their own behaviors [24]. Because of this they may be open to exploring events or experiences in their own personal history or circumstances that could pose a risk for their child (i.e. through their parenting or mental or physical health), and they may be motivated to participate in interventions designed to reduce or even eliminate those risks. Moreover, intervention programs that act early in the prenatal period, might offer the greatest potential for both effectiveness and future cost savings over the entire life course [25, 26].

A significant body of literature exists examining perinatal interventions to improve broad child health outcomes. However, to our knowledge, there are few reviews that specifically consider ACEs preventive interventions during this life stage, and none that apply the public health prevention and life course frameworks or focus on rigorously designed experimental studies. To address this gap in the literature, we undertook a scoping review of perinatal interventions designed to address at least one risk or exposure related to child ACEs. Our aims were (1) to conduct a systemic search of published perinatal interventions tested in RCTs with the purpose of preventing or addressing ACEs and their risk factors and had reported results for mother and child; (2) to assess the degree of potential bias, transparency, and reproducibility of the reported study designs and evaluation procedures; (3) to perform content analysis using (a) the public health prevention continuum and (b) life course intervention characteristics framework to describe where the interventions lie along the prevention continuum and the degree to which they incorporate life course characteristics; and (4) to summarize which interventions demonstrate effectiveness at reducing the risk of future exposure of the child to ACEs and its sequalae.

## Methods

### Inclusion criteria

Given that the term ‘ACEs’ has only recently become widely used, and few interventions directly addressed ‘ACEs prevention’, studies were included in this analysis if they aimed to address risk factors or potential exposure for the future experience of an ACE by the infant. Search terms included all the named adversities in the landmark Felitti study (physical, sexual, or emotional abuse, physical or emotional neglect, parental mental illness, parental substance abuse, intimate partner violence, parental divorce, having a relative in jail or prison), as well as adversities such as experience of racism, stigma, discrimination, or prejudice; experiences of community violence; peer victimization and bullying; family conflict; and chronic social vulnerability including poverty and food insecurity [1, 27–30]. These broader adversities were included as they are increasingly recognized as stressors and traumatic experiences relevant to traditionally disadvantaged and marginalized groups and likewise associated with poor health outcomes [31–33].

Interventions were categorised as perinatal if they were begun during pregnancy and were substantially delivered during the time between conception and 4 months post-partum, the latter period often referred to as the “fourth trimester of pregnancy”, acknowledging it as a challenging time of adaptation for parents and child [34]. Interventions that initially engaged and intervened with mothers, infants, and families only after birth were excluded from this review. To focus on interventions tested with the strongest study design, this review was further limited to Randomized Controlled Trails (RCTs). To be most relevant to contemporary family and community conditions, we included only articles published between January 2000 and November 2023 in English. As we wished to focus on interventions that adopted at least a dyadic approach, this review was further limited to studies that reported outcomes for both mother and child.

### Search method

A research team was formed, and with the assistance of research librarians, a literature search was conducted in February 2022 with a supplemental search in November 2023 in Psychinfo and PubMed databases. ACEs did not exist as a MESH term until 2019, therefore, we used MESH terms and key words based on the ‘Improve Child Outcomes Associated with Adverse Childhood Experiences’ systemic review, to guide our search strategy [18]. The terms included were child maltreatment, early life trauma, child abuse, childhood trauma, abuse, neglect maltreatment or sexual violence, battered child mistreatment, partner violence, domestic violence, various mental health diagnoses, substance use disorders, incarcerated, divorced, separated parents, social discrimination, racial biases, and racism. We included primary, secondary, and tertiary prevention interventions regardless of their setting. We also reviewed reference lists of relevant evidence reviews for primary studies that fit the inclusion criteria.

**Fig 1:**
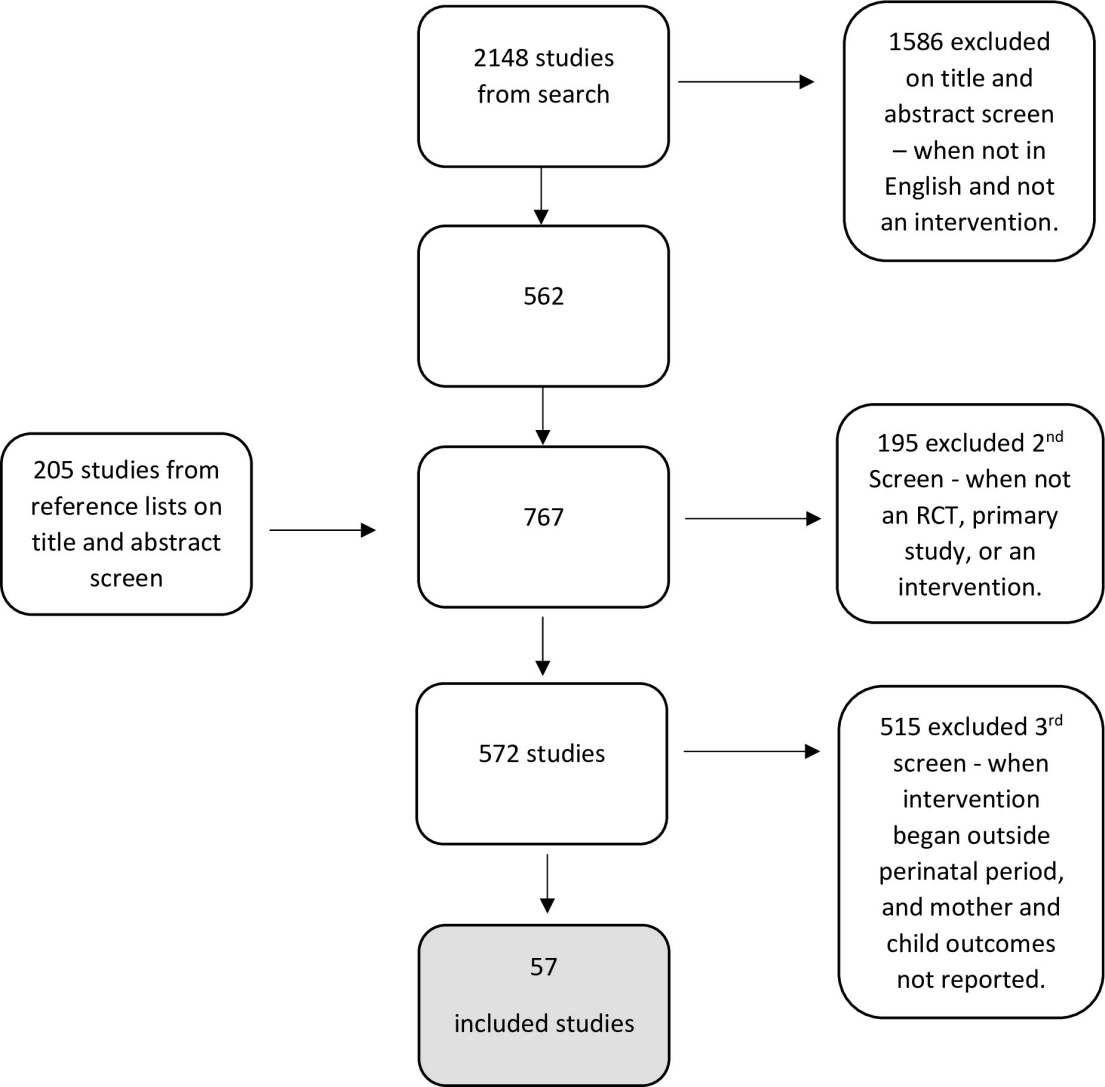
PRISMA chart outlining the flow of studies at each stage of the review.

We used an adaptation of the PRISMA-ScR process to determine which studies would be included in our full analysis [35]. (Refer to S1 Appendix, PRISMA-ScR-Checklist.) First, a single reviewer independently screened titles and abstracts for the 2148 studies identified through the initial search. Excluding duplicate articles, prevalence studies, opinion pieces, descriptive association studies, those that did not report results, and those not relevant to the topic excluded 1586 articles, leaving 562 under review. Review of reference lists led to identification of 205 additional candidate articles, yielding 767 for further review. Of these, 195 were deemed out of scope. (see “Fig 1”).

Once narrowed to 572 studies, three research team members independently read and classified the studies using the following categories: title, author, year of publication, type of study, the strength of the study (design, bias), description of population, description of intervention, and reported outcomes. At this step, articles were further excluded if they were not RCTs, not implemented during the perinatal period, and did not report outcome measures for both the mother and child. This left 57 articles which on further review yielded 53 separate interventions (4 were articles about interventions already included) for our final analytic sample of studies for deeper analysis.

### Data extraction

Three members of the research team reviewed the included studies and extracted data and descriptions according to the categories listed below. A summary of the key features of included articles were captured in a table. (Refer to S2 Appendix, Features of articles). Categories were selected based on relevance to the study aims and on the PRISMA-ScR checklist [36]. Discrepancies in observations between reviewers were resolved through dialogue to reach consensus.

1. Study description: author, year, title, source, abstract, study design, randomization method, blinding method of participants and researchers, allocation method, setting of intervention, geographical location, level of detail about the intervention provided.
2. Type of study: name of intervention, type of intervention, description of the intervention, the aim of the intervention, target of intervention, the intervenors, dose of the intervention
3. Participants: the number of participants and their race, ethnicity, language spoken, income level, educational level, relational involvement in intervention,
4. Impact and outcome: measures used, impact and outcome, length of follow-up, consideration of bias risk, and strength of study overall.

### Considerations of bias

The degree of study bias was evaluated by adopting the Cochrane Collaboration’s tool for assessing the risk of bias in randomized trials by Higgins et al. [37]. This tool incorporates six principles for assessing bias in randomized controlled trials. The bias domains included: Selection bias (1. Random sequence generation, 2. Allocation concealment), Performance bias (3. Participant and 4. Personnel blinding), Detection bias (5. Outcome blinding), and Attrition bias (6. Incomplete outcome data). Studies were classed as having a low risk of bias if each domain was classified as low risk and the presence of bias was unlikely to alter the results seriously; a high risk of bias if at least one of the domains was classified as having a high risk of bias and the bias may alter the results seriously, and an unclear risk if the authors were not able to gather enough information from the written reported process about the details of the bias control process. Two evaluators worked together to assign categories and resolve any discrepancies, with a final assignment based on consensus. For a summary of this process refer to S3 Appendix, Critical Appraisal.

### Transparency and reproducibility of intervention content

We also evaluated the level of detail provided in each study publication to assess the study’s transparency about the content of the intervention, and to determine whether sufficient detail was provided, or available, to allow for reproduction, or spread and scale of the intervention. Papers with sufficient detail to understand what occurred at each step of the intervention were classed as ‘details were provided’. If the team were unsure about what occurred, or unable to reproduce an intervention, ‘limited detail reported’ was noted. These categories were based on current intervention reporting guidelines [38].

### Further content analysis

For each of the 57 articles, the team performed further analyses utilizing two frameworks: the Public Health Prevention continuum and the Life Course Intervention Research Framework [8, 20]. Consideration of the intervention for place on the public health prevention continuum was undertaken to determine the focus and emphasis of existing ACEs prevention research and to identify gaps. When applying the public health continuum to this review, primary prevention- focused interventions were defined as those designed for healthy women with no identified risk factors that may contribute to their child being exposed to ACEs; secondary as those offered to families at risk of exposing their child to ACEs e.g., a prior history of maternal depression, or history of substance use in remission at risk of relapse; and tertiary were those where an ACE exposure was identified (e.g. ongoing maternal substance use) and interventions were focused on eliminating, reducing the impact of and/or mitigating against the adverse exposure to the child. Each intervention was categorized according to its place along the continuum.

Consideration of Life Course Intervention Research (LCIR) Framework was also undertaken to determine to what degree existing ACEs prevention efforts incorporated a life course perspective. This framework is a useful tool to use since SDOH and the Socio Ecological Model are both built into this framework, because they are recognised as strong contributors to perinatal ACEs due to their impact on maternal physical and mental health. The LCIR framework is also helpful, given that ACEs themselves have impacts across the whole life course, and potential for intergenerational effects. The Life Course Intervention Research Network (LCIRN) recently undertook a modified Delphi process to delineate characteristics of life course interventions [20]. This process yielded an initial list of 12 characteristics. Life course interventions are (1) aimed at optimizing health trajectories; (2) developmentally focused, (3) longitudinally focused, and (4) strategically timed; and are (5) designed to address multiple levels of the ecosystem where children are born, live, learn, and grow and (6) vertically, horizontally, and longitudinally integrated to produce a seamless, forward-leaning, health optimizing system. Interventions are designed to (7) support emerging health development capabilities; are (8) collaboratively codesigned by transdisciplinary research teams, including stakeholders; and incorporate (9) family-centered, (10) strengths based, and (11) antiracist approaches with (12) a focus on health equity. These characteristics can be incorporated at all stages of an intervention, from conception and planning, to design, implementation, evaluation, and translation, with the whole comprising a Life Course Intervention Research (LCIR) Framework [39].

It has been suggested, but not proven, that interventions that adopt these characteristics may be more effective in having a positive impact on health. As a preliminary test of this theory, we analyzed to what extent interventions incorporated these characteristics, and whether interventions that incorporated a greater number of characteristics were more likely to show positive impacts on maternal and/or child outcomes. The number of life course characteristics included in the intervention design were determined for each intervention by two research team members, blinded to each other, and discrepancies resolved with a discussion between three team members until consensus was reached. To guide this scoring, the research team met and discussed the discrepancies and refined operational definitions of how each characteristic can be applied to interventions in the perinatal period. If the member’s score differed by three or less, the average was taken. For those that differed by more than three, the operational definitions were re-visited and refined, and then all three members re-scored the study independently. The re-score resulted in all studies having a less than 3-point difference among the three-member team. The operational definitions of the LCIR characteristics developed for this review were adapted by the authors, from the descriptions provided in Russ et al, 2021 [20]. (Refer to S4 Appendix, LCIR Characteristics.)

To understand the relationship between the number of incorporated life course characteristics and the outcomes reported for the mother and/or child, we performed a two-tailed t test on the equality of means, assuming unequal variances between the number of incorporated life course characteristics and whether the study reported a positive outcome for the mother and/or child. To evaluate for a threshold effect, we dichotomized the number of characteristics documented using a threshold of seven characteristics (i.e., 6 or less characteristics = low level of LCIR characteristics; 7 or more characteristics = high level of LCIR characteristics) based on the empiric distribution. We used a Fischer’s exact chi square to test the association between seven or more LCIR characteristics and whether the study reported a positive outcome for the mother and/or child. As robustness checks, we also used a threshold of 6 and 8 characteristics.

## Results

In-depth analysis of the 57 articles identified 53 distinct interventions, with 4 articles being follow up studies of the same intervention with the same population cohort. The RCTs were evenly distributed across the study extraction dates, with 37% between 2000 and 2009, 47% from 2010 to 2019, and 16% published since 2020.

### Quality of studies

No studies were identified with a high risk of bias, so none were excluded due to bias concerns.

### Study location

The interventions analyzed were from a wide range of countries. Less than half (40%) studies were from the United States, 15% each from the United Kingdom and Australia, 8% from other European countries, 6% from South Africa and China, and one each from Iran, Pakistan, Northern Ghana, Malaysia, India, and Nigeria. Most interventions were conducted in higher income countries (HIC) (83%), with 17% from a subset of Low- and Middle-Income Countries (LMICs) in the Middle East, Africa, or Asia.

### Study setting

Just over half (53%) of the interventions were home-based, 21% took place in a clinic setting, 17% in a community setting, and 4% were a combination of home and clinic based. Six studies incorporated a virtual component (phone or internet-based).

### Subject of intervention

The focus of the intervention varied; 43% worked directly with the individual pregnant woman, 38% had a focus on the mother-infant/child dyad, and 23% involved more than the mother/child dyad. Three interventions (19%) reported they involved the family unit, with seven studies (13%) who had a focus on involving co-parents / couples.

### Participants

#### Demographics

More than 20,000 pregnant women were participants in the 53 included studies. The demographic data of the participants were inconsistently measured and reported across the studies. Three interventions engaged indigenous minoritized groups, including American Indian and Native Hawaiian. Latinx was referenced in many studies as Hispanic. For the purposes of this article the authors will refer to Latinx or Non-Latinx in reference to an individual reporting or not reporting an ethnic heritage from a Latin American country. Of the 24 articles reporting studies from United States of America, 5 articles did not report ethnicity and/or race of participants. Of the 19 articles that identified ethnicity, four (from three separate interventions) identified American Indian or Hawaiian native ethnicity, ten Latinx participants, and six studies had majority African American/Black participants. None of the studies had either a majority or a large proportion of Asian/ Pacific Islander participants.

#### Spoken language

Three quarters of the studies indicated that they provided the intervention only in their national primary language. 16 interventions were offered in the predominant local language plus at least one additional language. Languages represented included English, Chinese, Viennese German, Yoruba, Persian, Dutch, Danish, or Malay, Spanish, indigenous languages, and local dialects in South Africa, Pakistan, and India.

### Adversities

Studies aimed to address a wide variety of traumas, adversities, or risk conditions, frequently addressing more than one risk. The adversities were grouped into eight broad categories which are not mutually exclusive. “Table 1” lists the number of interventions that targeted these risks or adversities.

**Table 1:** Adversities addressed across interventions.

Potential ACE exposure or ACE risk factor for the child targeted by the intervention	# (%) of interventions that targeted these risks or adversities
Social vulnerability e.g., low income, low parental education attainment	27 (51%)
Maternal perinatal depression or anxiety diagnosis and/or symptoms	16 (30%)
Healthy pregnant women with a focus on the prevention of Post-Partum Depression (PPD)	10 (19%)
Risk factors identified for child maltreatment	7 (13%)
Parent(s) is an adolescent	5 (9%)
Current or previous significant trauma and/or PTSD experienced by the parent	3 (6%)
Parents with identified ACE exposure	3 (6%)
Parental substance use, including smoking, alcohol, or other substances	2 (4%)

### Outcomes

Various outcome measures were used across studies, 76 for mothers and 41 for children. The most common maternal measure was the EPDS (Edinburgh Postnatal Depression Scale), used in 39% of the studies, with each version validated for its country of study. “Table 2” lists the tools used across the studies to measure maternal outcomes and “Table 3” lists tools used to measure child outcomes.

**Table 2:** Tools used to measure maternal outcomes.

Measure name	Description of measure	Measure name	Description of measure
EPDS	Edinburgh Postnatal Depression Scale	HFIAS	Food insecurity
PSI-SF	Parenting Stress Index short form	COPI	Mother-child interactions
Questionnaires	Patent satisfaction, general health, social support, craving	SF-36	quality-of-life measures
HOME	Quality home environment inventory	CTS-PC	Conflict Tactics Scale–Parent Child
STAI	State-Trait Anxiety Inventory	RAND MH	Mental health inventory for adolescents
STAXI	State-Trait Anger Expression Inventory	PSSS	Post-partum support
MINI	Mini-International Neuropsychiatric Interview	Perlin Mastery Score	Extent an individual regard their life chances as being under their personal control** **
BDI	Beck Depression Inventory	Self-report	Stress and mental health
BAI	Beck Anxiety Inventory	PSS	Perceived Stress Scale
CARE index	Maternal sensitivity/measure mother-child interaction	CDC scale	Depression and anxiety
WBPB	What Being a Parent of a Baby is like	POMS	Profile of Mood States
Qualitative interviews	Life history, recall birth outcomes, IPV, maternal smoking, daily hassles, sleep disturbance, fatigue	VITAS	Measure of Pain
Observational measures	In-person and using video, parenting practices, attachment, maternal interaction behavior	Lab Measures	Urine samples, nicotine use, cortisol levels, saliva
AAPI-2	Adult-Adolescent Parenting inventory	CES-D	Depression
MOS	Health-related quality of life–physical and mental health	DUFSS	Social support
EQ-5D	A measure of health-related quality of life	CAGE and ASI	Substance and Addiction
PHQ-9	A measure of the degree of depression	OHQ	Happiness
HRSD	Hamilton Depression Rating Scale	GAD-7	General Anxiety Disorder
HRSA	Hamilton Anxiety Rating Scale	GHQ-12	Psychological distress
MH15	Anxiety and depression	EAS	Parent-infant interaction
MAAS	A measure of trait mindfulness	SCS	Self-compassion Scale
WHO-DAS	World Health Organization Short Disability Assessment Schedule	DISC-12	Discrimination and Stigma
MAMA	Adjustment to pregnancy	SICS	Self-efficacy infant care
DSM-IV	Diagnostic and Statistical Manual of Mental Disorders, fourth edition	MSES	Self-efficacy
PCIS	Parent involvement scale	CSEI	Coopersmith Self-esteem Inventory
CESD–mod	Modified Parental wellbeing	MSRI	Self-esteem inventory
HITS scale	hurts, insults, threats, screams scale	PI	Predictive Index PPD
SOS	Significant Others Scale	WDW	Who Does What
PSOC	Parenting sense of confidence	CTS2	Interpersonal Violence
CR	Couple relationship	Medical record	Obstetric outcomes, Adverse pregnancy outcomes
PA	Partner awareness scale– 25 items	DASS	Depression Anxiety Scale
ASI	European Addictions Severity Index	WWS	Wang Withdrawal Scale
DRM	Day Reconstruction Method	HFIAS	Household Food Insecurity Access Scale
State admin records	Government assistance–Food stamps, AFDC (Aid to families with dependent children), TANF (Temporary Assistance for Needy Families)	AUDIT-C	3-item alcohol screen that can help identify persons who are hazardous drinkers or have active alcohol use disorders
RHI	Relational Health Index–Mentor Scale	RQ	Relationship Quality scale
FFMQ	Five Facet Mindfulness Questionnaire	BMSWB	Body-Mind-Spirit Wellbeing Inventory
SFS	Smilkstein’s Family System Apgar Items	PASS	Perinatal Anxiety Screening Scale
EAS	Emotional Availability Scale	ANRQ	Antenatal Risk

**Table 3:** Tools used to measure child outcomes.

Measure name	Description of measure	Measure name	Description of measure
CAPI	Child Abuse	WPPSI-IV	Preschool and primary intelligence
ASQ:SE-2	Social and emotional development	Griffiths score	Rate of infant development
Interaction	Scales used to assess child’s measure of hunger and talking to others, attunement	MIRI	Infant responsiveness
CBCL	Child behavior	PBI	Bonding
CPS	Child Protection Services records	MDI	Child Language
Observations	In-person or on video, Infant feeding, and sleeping, attachment security to mother, Child health development	APGAR	Appearance, Pulse, Grimace, Activity, and Respiration–a measure of baby’s condition immediately after birth
Child responsiveness	Infant Behaviour Questionnaire	Teacher’s reports	School performance
Finnegan scores	Quantify and diagnose neonatal withdrawal or abstinence (NAS) syndrome	EAS	Emotional Adaptability, Sociability, Temperament survey for children
Access to care	Well-child visits	NMR	Neonatal Mortality Rate
Measures	Infant height, length, weight, Fetal Activity/movement, fetal heartbeat, head circumference	MacArthur	Communicative development
SSP	Attachment Behaviour	ITSEA	Infant-toddler social and emotional assessment
PTB/VPTB	Pre-Term Birth, Very Pre-Term Birth	LBW/VLBW	Low Birth Weight, Very Low Birth Weight
CTP-S	Closeness to Parent Scale	SCAS	The Spence Children’s Anxiety Scale
Qualitative	Baby day diary	Executive function	Battery of tests
SDQ	Prosocial behaviour	Stroop-like silly sounds	Executive function 4-year-old children
Bayley scales	Infant and Toddler development	Aggressive behaviour	A subscale of Child Behaviour Checklist
Kaufman Assessment	Battery of tests	Medical records	Neonatal outcomes
Child Protection records	Injury or concern of abuse or neglect in first 24 months	CTS-PC	Conflict Tactics Scale–Parent Child
NDKQ	Newborn Developmental Knowledge Questionnaire	CIN	Child In Need–as recorded by children’s social care services
Early childcare and education records	Attendance	School readiness and attainment	At key stage 1
Administration	Healthcare costs		

### Impact

Overall, from the 57 articles 42 (74%) reported a positive impact on mother and/or child.

- 37 (65%) reported positive outcomes for mothers
- 37 (65%) reported positive outcomes for children,
- 32 (56%) reported positive outcomes for both mother and child
- 15 (26%) interventions reported no significant impact on either the mother or child

“Table 4” indicates the different intervention types and “Table 5” indicates the discipline of the person who delivered the intervention.

**Table 4:** Reported impact on the mother and/or child by the intervention type.

Intervention approach	# (%) interventions	Positive impact on mother and/or child	No significant impact on mother or child
Integrative health	5 (9%)	4 (80%)	1 (20%)
Psychological interventions	124 (23%)	10 (83%)	2 (17%)
Home-visiting program	27 (51%)	17 (63%)	10 (37%)
Education	7 (13%)	4 (57%)	3 (43%)
Virtual delivery	5	4 (80%)	1 (20%)
Medication and supplements	2 (4%)	-	2 (100%)

**Table 5:** Reported impact on mother and / or child by discipline of the intervenors.

Intervention providers	# (%) of interventions involving the listed providers	Positive impact on mother and/or child	No significant impact on mother or child
Social workers	1 (2%)	1 (100%)	-
Personal trainers or instructors e.g., massage, yoga	4 (8%)	4 (100%)	-
Mental health clinicians e.g., psychologists, counselors, psychotherapists	14 (26%)	12 (86%)	2 (14%)
Child health or public health nurses	16 (30%)	11 (69%)	5 (31%)
Paraprofessionals	8 (15%)	6 (75%)	2 (25%)
Midwives, obstetric nurses	4 (8%)	2 (50%)	2 (50%)
Peers or volunteers with training	8 (15%)	4 (50%)	4 (50%)
Physicians e.g., psychiatrists, obstetricians	3 (6%)	1 (33%)	2 (67%)

“Table 6” below categorizes interventions based on content and the ACEs risk factor they were designed to address.

**Table 6:** A summary of the type of interventions that were provided to address ACE risk factors.

Number interventions (%)	Brief Description of type of intervention	Names of the interventions delivering	ACE risk factor being addressed
17 (32%)	Structured program involving nursing visits in the home	• Nurse Home Visit (HV) • Nurse Family Partnership (NFP) • Group Nurse Family Partnership (g-NFP) • Healthy Families (HF) • Dads matter-HV	• Antenatal depression • Social vulnerability • Parental ACEs • PTSD/trauma exposure • Adolescent Parents • Addictions
11 (21%)	Home visiting program delivered by paraprofessionals	• Healthy Start Program • Paraprofessional Home Visit • Mentor-mothers • Dads Matter-HV	• Social vulnerability • Prevention Postnatal depression
6 (11%)	Taught massage and or yoga in a class. Audio sound for relaxation	• Infant massage • Massage • Yoga • Audio relaxation • Thriving in Pregnancy–Cultivating the Four Immeasureables.	• Prevention postnatal depression
5 (9%)	Dyadic psychological intervention to support infant attachment and bonding	• PREEP • Parent-infant psychotherapy • Newborn Behavioural Observations	• Parental ACE • Social vulnerability • Existing MH diagnosis
4 (8%)	Education program Could be online or in a structured education program	• Internet support program • WWWT (What Were We Thinking) • Ready for Child Program • MBSP (Mindful Self-compassion Program)	• Prevention Postnatal depression
3 (6%)	Antenatal cognitive behavioral therapy (CBT) provided by trained therapists	• Cognitive Behavioral Therapy	• Prevention Postnatal Depression • Reduce Interpersonal violence • Reduce perinatal depression
2 (4%)	Peer delivered Cognitive Behavioral Therapy (CBT)	• IMBC–Integrated Mothers and Babies Course • Thinking Healthy Program	• Prevention Postnatal Depression
2 (4%)	Co-parenting classes that offer brief intervention prevention programs to enhance parent coordination and parenting roles	• Family Foundations	• Prevention Postnatal Depression, Anxiety and Stress
1 (2%)	Efficacy buprenorphine or methadone	• Medication	• Opioid Addiction
1 (2%)	Regular supplement during pregnancy	• DHA Supplement	• Prevention Postnatal Depression
1 (2%)	Community participatory women’s groups to support establishment of social support and networks	• Participatory intervention women’s groups	• Prevention Postnatal Depression

“Table 7” lists the number of studies with certain follow up periods within their study and also identifies whether they had a positive or negative impact on the mother and/or the child.

**Table 7:** Follow up period.

Follow up period	# (%) studies	Positive impact on mother and/or child	No significant impact on mother or child
< or at 4 months follow up	18 (32%)	15 (83%)	3 (17%)
4 to 12 months	18 (32%)	12 (67%)	6 (33%)
>1 to 2 yrs.	6 (11%)	4 (67%)	2 (33%)
>2 yrs.	15 (26%)	10 (67%)	5 (33%)

In terms of longitudinal follow-up, there were 42 studies (74%) that measured outcomes beyond the 4-month post-partum period. Nine of the fourteen interventions that reported follow up for two years or more reported a sustained positive impact on the mother and/or child. Almost all of these were home visiting programs, apart from one participatory intervention community-based woman’s group. Most interventions (63%) had periods of follow-up of 12 months or less, therefore that their long-term or life course impacts are unknown.

### Content analysis used in studies

See “Table 8” for the distribution of interventions across primary, secondary, and tertiary prevention categories.

**Table 8:** Interventions by public health prevention continuum domain.

Public health Prevention domain	Number interventions	Positive impact mother and/or child	No significant impact mother or child
**Primary**	10 (19%)	8 (80%)	2 (20%)
**Secondary**	28 (53%)	19 (68%)	9 (32%)
**Tertiary**	15 (28%)	12 (80%)	3 (20%)

“Table 9” describes the number and percent of interventions that incorporated each of the LCIR characteristics.

**Table 9:** Incorporation of LCIR characteristics into interventions.

LCIR characteristics	Number and (%) of interventions incorporating this element into the design
Strategically timed	53 (100%)
Developmentally focused	53 (100%)
Longitudinal focus–Follow up 6 months or longer	35 (66%)
Emerging health development capabilities	35 (66%)
Multi-level/Holistic	35 (66%)
Health equity focused	24 (45%)
Family-centered	23 (43%)
Health optimization	23 (43%)
Strengths-based	15 (28%)
Vertically, Horizontally and Longitudinally Integrated	13 (26%)
Collaboratively co-designed	2 (4%)
Anti-racist	1 (2%)

**Fig 2:**
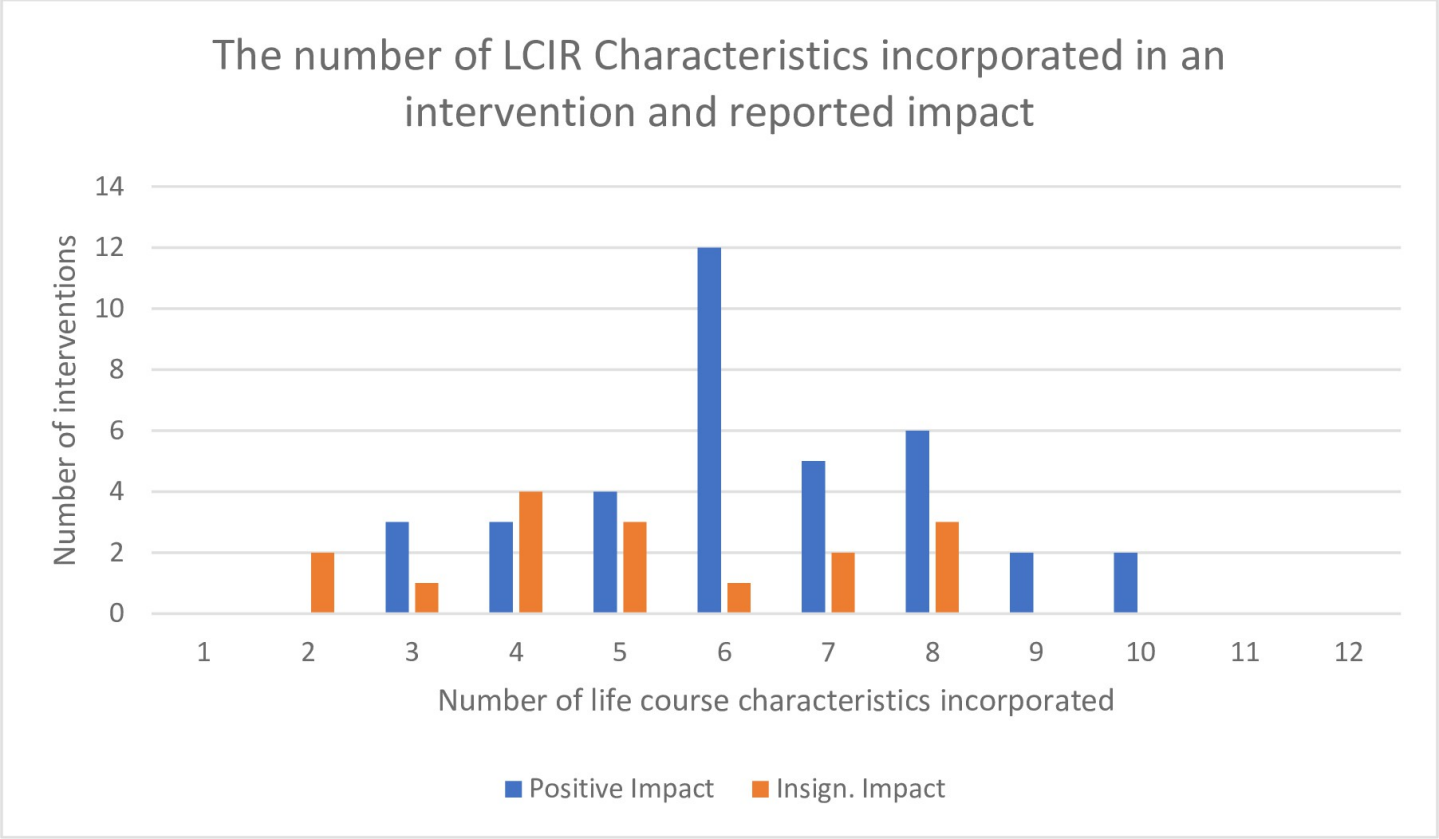
The number of LCIR characteristics incorporated in an intervention and reported impact.

“Fig 2” demonstrates the number of interventions that incorporated various levels of LCIR characteristics into their design. All 53 interventions were developmentally focused and strategically timed to a sensitive period of brain development, as they were all implemented during the peri-natal period. Over half incorporated a longitudinal focus, targeted emergent health development capabilities, and were multi-level. No interventions incorporated all LCIR characteristics into their design. Characteristics that were less frequently incorporated included a focus on health equity, being family-centered, explicitly anti-racist (only 1(2%)), integrated with other services, and collaboratively co-designed with family and community representatives and with potential intervention recipients (Only 2 (4%)). Fathers were actively involved in the intervention in only 13/53 (25%) studies.

Of the 14 interventions which reported no significant impact for mother or child, all 14 lacked collaborative co-design and specific anti-racist approaches, 10 did not incorporate family-centered design or service integration characteristics, nine did not include emerging health development capabilities, seven did not include holistic or multi-level characteristics, six did not have an equity characteristic and two did not report longitudinally focused characteristics.

There were 33 interventions (62%) that incorporated up to half (six or less characteristics), and of these studies, 67% had a positive impact on the mother or child. Of the twenty interventions that incorporated seven or more characteristics, 75% reported a positive impact and 25% reported insignificant impact on mother and or child.

The mean number of LCIR characteristics was significantly higher in the studies that reported a positive impact on the mother and/or child (p = 0.003). We found a significant association between the number of life course characteristics incorporated in an intervention and whether the study reported a positive impact on the mother and/or child at a threshold of six (p = 0.004) and eight (0.04), but not seven (p = 0.07) life course characteristics.

## Discussion

Preventing Adverse Childhood Experiences (ACEs) is recognised as a public health priority. The perinatal period offers a compelling opportunity for effective prevention efforts to optimize children’s health development trajectories and break intergenerational cycles of adversity. We analyzed 53 interventions that occurred in the perinatal period with a majority focused on secondary prevention. We found that most studies reported some positive impact for mother or child. Although most interventions were focused at an individual level, the authors recognise the impact that structural SDOH factors have on perinatal ACEs, and understand that the incorporation of the Socio Ecological Model into the design of interventions would help to address this. The authors used the LCIR framework, which has the Socio Ecological Model built into it, to determine the extent to which it was incorporated within the interventions themselves. We found that there was a significant association between the number of life course characteristics incorporated in an intervention and a reported positive impact on the mother and/or child. Few interventions incorporated community context or wider family involvement, and very few considered adversities such as racism and community violence.

This scoping review contributes to an ongoing knowledge synthesis that summarizes the state of ACEs prevention research and starts to identify the most effective strategies that can be applied both to at-risk and general populations. We found that few perinatal interventions were specifically aimed at preventing ACEs as a broad set of adversities, but many were designed to prevent and/or address individual adversities, and risk factors for adverse experiences. While it is useful to identify preventive interventions for specific risks, in practice ACEs rarely occur in isolation, and frequently cluster. This signals a need for more intervention research that targets a range of risks. Seventy- percent of RCTs we reviewed demonstrated positive impacts on the child and/or mother, suggesting the perinatal period may be a promising stage for ACE prevention intervention.

### Longitudinal impact

Expectant parents can be highly motivated to make changes to benefit their unborn child, providing an ideal time during the life course for community programs to embrace this opportunity [22, 40, 41]. Yet, this potential is easily squandered if the interventions offered to them are not informed by the evidence base, are too narrowly defined, or fail to meet family and community needs. Ideally, ACEs preventive interventions would have long-lasting impacts that go beyond simple time-limited risk reductions and instead improve life course health trajectories. Given that only approximately one-third (37%) of studies followed up for more than one year, and only 26% for more than two, it is not possible to determine the long-term impacts of most of these interventions. However, of the interventions that did follow up for two years or more, 67% reported sustained positive impact, an encouraging finding. While this may reflect some bias whereby better-funded studies with more intense interventions were also more likely to be able to follow participants for longer time periods, and that long-term follow up studies with positive findings may be more likely to be submitted and published than those with negative findings it could also suggest that these more comprehensive interventions may indeed have longer-lasting impacts.

### Home visiting interventions

Nurse-family partnership home visiting programs are by far the most intensively studied perinatal intervention related to ACEs prevention. While many home visiting interventions are based on the Olds model [42, 43] there is considerable variation in content and delivery, including whether the intervention is led by nurses, paraprofessionals, or volunteers. A large body of literature has yielded mixed results on their effectiveness, efficacy, and potential for spread and scale [44]. The Home Visiting Evidence of Effectiveness program maintains a website summarizing the state of the evidence for different models (https://homvee.acf.hhs.gov/). In turn, there have been tensions about whether to prioritize fidelity to original models, or to emphasize the need for sometimes significant cultural and local adaptations [45]. These difficulties have been compounded by the challenges inherent in determining which components of the program are essential vs flexible, and the need for greater detail in reporting on the content and activities undertaken during visits for different populations [46, 47]. In our review, 63% home visiting interventions reported positive impacts, but more than one-third did not, a high proportion when one considers the resources applied to home visiting. Only 20 (35%) incorporated 7 or more life course characteristics into their design, only 13 (26%) involved family members other than the child’s mother, 2 showed any evidence of any co-design, and only 1 study reported incorporation of anti-racist principles. Adaptations to home visiting programs that incorporate these elements could improve impact and are worthy of further study.

The higher likelihood of a reported positive perinatal intervention outcome when more life course characteristics were reflected in the intervention design suggests that greater incorporation of life course characteristics in general, beyond just home visiting programs, merits further study. For example, it would be of great interest to know whether incorporation of all 12 characteristics could result in interventions having even greater positive dyadic impacts that were sustained across the life course. One intervention that incorporated 10 characteristics was the secondary prevention-focused indigenous Family Spirit program, with strong culturally led equity-focused co-design, that reported sustained (3 years) positive impacts on mother and child [48, 49]. Characteristics least likely to be incorporated in current interventions include some identified by stakeholders as most important for life course orientation, including strengths-based approaches, a focus on health optimization, collaborative co-design and incorporation of specific anti-racist components [20].

### Interventions in the context of the developmental ecosystem

Life course approaches acknowledge that ACEs occur and exert their effects in the context of the child’s family, neighborhood, social and community environment, yet very few studies in this review either reported on or considered in depth this broader developmental ecosystem. Most studies focused on the mother alone, some included the child or infant and only 13% included the child’s father or second parent or any other family members. This approach represents a missed opportunity for wider family and community engagement in ACEs prevention efforts that may have limited intervention effectiveness [50]. Co-parenting interventions such as Family Foundations could prove particularly impactful for ACEs prevention yet are not in widespread use [51]. Similarly, a lack of co-design, present in only 4% interventions, represents a missed opportunity for individuals and families to contribute their own lived experience and knowledge of local context to the development of culturally and contextually relative interventions created in equal partnership. Only one quarter of interventions considered individual, family and community strengths, representing a missed opportunity, and also pointing to a need for more detailed research on definitions, principles and use of different types of strengths of character, skill, and resources [52]. These omissions also point to a gap in research for new approaches to family and community engagement in intervention studies to better understand priority needs.

Only 1 of the 53 interventions in this review specifically addressed racism as a root cause contributor to adversity. This critical omission suggests that current intervention approaches are not considering the role of structural factors and biases in society and institutions that can contribute to early adversity and reinforce inequity. Greater incorporation of equity aims and anti-racist approaches into intervention design and implementation could go some way to ensuring root causes of adversity and trauma are acknowledged [53].

Finally, despite good evidence for short and medium-term positive impacts of a range of perinatal interventions, each intervention on its own addressed the prevention of only certain adversities. One option for future research is to integrate or “bundle” together multiple interventions, or core components of evidence-based interventions into larger, or stacked multi-level interventions, linking them more overtly with existing programs to address multiple co-existing needs [54, 55]. This approach has proven promising in early childhood interventions to promote early learning, but requires rigorous study in relation to ACEs prevention [56].

### Limitations

Limitations of this scoping review include our choice of search terms and inclusion criteria which inevitably impact the articles under review, and our limitations on detailed understanding of the intervention content, intensity, and activities to the detail reported in the manuscripts. This review did cover a select population due to the fact that we included only articles published in English, and most reported. most interventions were delivered in English and 1 or at most 2 other languages. Therefore, it is uncertain to what degree our findings might be relevant for non-English speaking countries, groups and cultures. All review elements were limited to manuscript content–for example if authors chose not to publish negative impact or lack of impact of interventions this could not be considered in our review. Due to the imbalance in the number High Income Countries (HIC) compared with Low and Middle Income Countries (LMIC) comparisons between them were not conducted. This means our findings cannot be generalized to non-English speaking countries and to LMICs.

The two conceptual frameworks driving this review, the public health prevention continuum, and the Life Course Intervention Research framework, gave useful insights, but different conclusions may have been drawn from use of other frameworks. Moreover, the finding regarding life course characteristic incorporation and parent/child outcomes should not be interpreted as causal as there is a potential for unmeasured confounding.

Interventions were classed as having positive impact if the statistical analysis reported in the manuscript supported this conclusion, however few authors commented on the degree to which significant findings were clinically meaningful or were classed as meaningful in a practical sense by the intervention recipients themselves. Incorporation of standardized measures across research on ACEs prevention could facilitate cross study comparisons. The authors encourage greater attention to the sharing of intervention content and design processes globally to increase learnings across the field.

### Future directions

There were a limited number of longitudinal studies identified for this review therefore there was limited ability to understand the impact on adolescents and adults across the lifecourse from the interventions. The authors support further longitudinal research, both to better understand the pathways from ACEs to health outcomes later in the life course, and to evaluate the impact of perinatal prevention efforts beyond childhood through adolescence, adulthood and later motherhood and fatherhood.

The imbalance between the number of studies conducted in HIC, compared with LMIC made comparisons difficult. The authors encourage further research to gain an understanding of the effects of WEIRD (Western, Educated, Industrialized, Rich and Democratic) contexts on intervention design and effectiveness.

The authors suggest that the utilization of the LCHD framework by researchers, practitioners and policy makers would be helpful to guide implementation efforts and help to shift the focus from individual to more multi-level interventions and can help to guide investment further upstream to prevent perinatal ACEs. Although the LCHD Framework was used to give consideration of the impact of SDOH by the incorporation of the Socio Ecological Model, more research is needed to better understand the evidence base for interventions that are multi-level and target broader structural issues.

### Public health implications

The perinatal period holds promise as a life course stage when public health programs targeting ACEs preventive interventions can have a positive impact on the future health of both mother and child. Existing service delivery programs that already work with perinatal populations e.g. home visiting programs could incorporate additional evidence-based ACEs prevention interventions identified in this review, including co-parenting and integrative health interventions.

Bundled multi-level evidence-based interventions that address social and structural determinants of health in their design and delivery could form a more comprehensive perinatal ACEs prevention strategy. Incorporation of life course characteristics including co-deign of interventions with potential recipients, and incorporation of trauma-informed elements into interventions is a strategy worthy of future public health research.

## Conclusion

This scoping review aimed to summarize the evidence from randomized control trials of interventions relevant for ACEs prevention delivered during the perinatal period, with measures for both maternal and child health outcomes, to understand the state of the evidence-base and the types of interventions being studied, as well as to start to elucidate the critical elements of interventions that significantly impacted mother and/or child. No interventions published within the scope of this review addressed ACEs per se. Instead, they targeted specific risks or conditions that placed the child at risk of ACE exposure. None of the interventions studied were sufficient as standalone single solutions to prevent and mitigate the impact of ACEs in the perinatal period and achieve transformative change. There is no ‘quick fix,’ and no ‘one size fits all’. Programs that incorporate multiple elements of successful interventions with delivery customized for different family and community contexts in flexible, adaptable ways may help prevent multiple ACEs. Incorporation of co-parenting interventions into home visiting models with wider adaptation to family and community context could be particularly useful.

Viewing ACEs prevention from a Life Course perspective provides an opportunity to guide design efforts to intervene in the perinatal period to prevent or reduce the life- long impact of ACEs. Preliminary analysis suggests that when more life course characteristics are built into interventions there is higher likelihood of positive outcomes. This review urges further efforts to understand the relative importance and impact of life course characteristics for interventions designed to prevent ACEs and improve life course health trajectories for mothers, children, families, and communities.

## Supporting information

S1 Appendix PRISMA-ScR-checklist. (PDF)

S2 Appendix Features of articles. (DOCX)

S3 Appendix Critical appraisal. (DOCX)

S4 Appendix LCIR characteristics. (DOCX)
