# Supplementary material for: Perinatal interventions to prevent Adverse Childhood Experiences (ACEs): A scoping review
Source: PLoS One. 2024 Oct 24;19(10):e0307441. doi: 10.1371/journal.pone.0307441 (PMC11501017; doi:10.1371/journal.pone.0307441)
Supplement: S2 Appendix — (DOCX) [file pone.0307441.s002.docx]

Appendix 1 – Summary of key features of included articles.

| **Author, Year,**  **Country** | **Title of article** | **Number of participants enrolled brief description and language requirements** | **Number and type(s) of trauma being addressed** | **Study Aim** | **Description of study (Discipline of intervenor(s), target & setting of study)** | **Details of the interventions provided.** | **Length of follow-up** | **Strength study** | **Prevention continuum** | **# Lifecourse Intervention Characteristics analysis** |
| --- | --- | --- | --- | --- | --- | --- | --- | --- | --- | --- |
|  |  |  |  |  |  |  |  | **Impact of study** |  |  |
| 1  Armstrong et al  2000  Aust. | Promoting secure attachment, maternal mood, and child health in a vulnerable population: a randomized controlled trial. ^[[1]](#footnote-1)^ | 181 families, Intervention (90) and Control (91).  Vulnerable families with Parents with ACEs, and or presence of 3 or more current risk factors.  English only | Multiple | To assess the effectiveness of a home-based intervention, targeted to identified vulnerable families and focused primarily on promoting the quality of the parent–infant interaction, and on preventative health practices that were most likely to benefit the child. | Child health nurse | Details were provided and can be made available. | 4 months  post-natal | Strong design | Secondary | 7 |
|  |  |  | Parental ACE history  Social vulnerability  Prevention child abuse |  | Family unit |  |  | Insign. impact mother |  |  |
|  |  |  |  |  | Home |  |  | Sign. impact on child |  |  |
| 2.  Austin et al  2008  Aust. | Brief antenatal cognitive behaviour therapy group intervention for the prevention of postnatal depression and anxiety: a randomised controlled trial. ^[[2]](#footnote-2)^ | 277 women, intervention (191) and control (86).  Perinatal baseline level of minor depression  English only | Single | To examine whether a brief antenatal CBT group treatment program plus information booklet, compared to an information booklet alone control condition, would result in less depressive and anxiety symptomatology in the postnatal period. | CBT Psychologist | Details were provided. | 4 months post-natal | Mod | Secondary | 3 |
|  |  |  | Perinatal depression (not severe) |  | Group-based, pregnant women |  |  | Insign. impact |  |  |
|  |  |  |  |  | Clinic setting |  |  |  |  |  |
| 3.  Barlow et al  2007  UK | Role of home visiting in improving parenting and health in families at risk of abuse and neglect: results of a multicentre randomised controlled trial and economic evaluation. ^[[3]](#footnote-3)^ | 131 women, Intervention (67) and Control (64).  Vulnerable families, primarily white, low income, and low education.  English only | Multiple | To evaluate the effectiveness and cost effectiveness of an intensive home visiting programme in improving outcomes for vulnerable families. | Family Partnership  Nurse | Limited details provided | 12 months post-natal | Strong design | Secondary | 7 |
|  |  |  | Parental ACE history  Social vulnerability  Perinatal baseline MH symptoms  (not severe) |  | Mother-infant dyad |  |  | Insign, impact mother |  |  |
|  |  |  |  |  | Home |  |  | Insign impact child |  |  |
| 4.  Barlow et al  2013  US | Effect of a paraprofessional home-visiting intervention on American Indian teen mothers’ and infants’ behavioural risks: a randomized controlled trial. ^[[4]](#footnote-4)^ | 322 Pregnant American Indian adolescents  English and native language | Multiple | To examine the effectiveness of Family Spirit, a paraprofessional-delivered, home-visiting pregnancy and early childhood intervention, in improving American Indian teen mothers’ parenting outcomes and mothers’ and children’s emotional and behavioural functioning 12 months postpartum | Family Spirit  Paraprofessional | Details provided | 12 months post-natal | Mod | Secondary | 10 |
|  |  |  | Adolescents  Social vulnerability  Rural residing on reservation |  | Mother and child |  |  | Sign. impact on mother |  |  |
|  |  |  |  |  | Home |  |  | Sign. impact on child |  |  |
| 5  Barlow et al  2015  US | Follow up of study above  Paraprofessional-delivered home-visiting intervention for American Indian teen mothers and children: 3-year outcomes from a randomized controlled trial. ^[[5]](#footnote-5)^ | As above | As above | The aims of this trial were to evaluate the intervention’s effects on parental competence (parenting knowledge, locus of control, stress, and behaviours) and maternal behavioural problems that impede effective parenting through early childhood (0 to 36 months postpartum). | As above | Details provided | 36 months post-natal | Strong design | As above | 10 - As above |
|  |  |  |  |  |  |  |  | Sign. impact on mother |  |  |
|  |  |  |  |  |  |  |  | Sign. impact on child |  |  |
| 6.  Barnes et al  2009  UK | The utility of volunteer home-visiting support to prevent maternal depression in the first year of life. ^[[6]](#footnote-6)^ | 1007 enrolled, Intervention (541) and Control (466).  Then matched 92 Intervention and 92 control and 66 no home-start.  Mostly White, low income and low education.  English only | Single | To evaluate the impact on maternal depression experienced in the first year of volunteer (Home-Start) support offered in late pregnancy or just after birth compared with families of similar vulnerability not receiving volunteer support. | Volunteers | Limited details provided | 12 months post-natal | Mod | Secondary | 4 |
|  |  |  |  |  | Pregnant women |  |  |  |  |  |
|  |  |  | Social vulnerability |  | Home |  |  | Insign. impact |  |  |
| 7.  Barnes et al  2017  UK | Randomized controlled trial and economic evaluation of nurse-led group support for young mothers during pregnancy and the first year postpartum versus usual care. ^[[7]](#footnote-7)^ | 166 enrolled, intervention (99) and control (67)  Mostly White, less than 24 yrs, low education  English only | Multiple | To determine the effectiveness and cost-effectiveness of gFNP (group Family Nurse Partnership) in reducing risk factors for maltreatment with a potentially vulnerable population. | Nurses | Limited details were provided | 12 months post-natal | Strong design | Secondary | 4 |
|  |  |  |  |  | Group, pregnant women |  |  |  |  |  |
|  |  |  | Adolescents and young  Social vulnerability  At risk child maltreatment |  | Clinic |  |  | Insign. impact |  |  |
| 8.  Bartu et al  2006  Aust. | Postnatal home visiting for illicit drug-using mothers and their infants: a randomised controlled trial. ^[[8]](#footnote-8)^ | 152 enrolled  Illicit Drug-using pregnant women.  Not reported - Likely English only | Single | To evaluate the effectiveness of six-month postnatal home visiting. The primary outcomes were duration of breastfeeding, and immunisation rates. Secondary outcomes were retention in the study and reduction in drug use. | Midwives | Details were provided | 6 months post-natal | Mod | Tertiary | 4 |
|  |  |  |  |  | Pregnant women |  |  |  |  |  |
|  |  |  | Perinatal Substance use |  | Home-visiting |  |  | Insign. impact |  |  |
| 9.  Baumgartner et al  2021  Nth Ghana | Effect of a lay counsellor-delivered integrated maternal mental health and early childhood development group-based intervention in Northern Ghana: A cluster-randomized controlled trial. ^[[9]](#footnote-9)^ | 32 clusters of women’s groups, 374 women, intervention (221) and control (153). Approximately 10 women per cluster  Pregnant women who lived rurally, low education, 39% with IPV.  Native languages | Multiple | To evaluate the impact of a group-based, cognitive-behavioural therapy intervention for women delivered during pregnancy and the postpartum period. (Integrated Mothers and Babies Course (iMBC)) | Peers  ‘model mothers’ | Details were provided | 19 months post-natal | Mod | Secondary | 6 |
|  |  |  |  |  | Mother and child |  |  |  |  |  |
|  |  |  | Rural  Social vulnerability |  | Community  Group |  |  | Insign. impact |  |  |
| 10.  Berry et al  2021  US | Perinatal depression prevention through the mother-infant dyad: The role of maternal childhood maltreatment. ^[[10]](#footnote-10)^ | 109 Latine women.  Low income and included with or without child maltreatment  English only | Multiple | This study describes the results of a secondary analysis of a combination of two randomized control trials (RCTs) of the Effective Postpartum Parenting (PREPP) intervention on the efficacy of preventing perinatal depression and optimizing infant regulatory behaviour, each outcome moderated by Childhood Maltreatment (CM). | Psychologist | Details were provided | 16 weeks post-partum | Mod | Secondary | 6 |
|  |  |  |  |  | Mother-infant dyad |  |  |  |  |  |
|  |  |  | Parental ACE history  Social vulnerability  Child maltreatment |  | Clinic based |  |  | Insign. for women and child with CM history |  |  |
|  |  |  |  |  |  |  |  | Sign. impact on mother and child if no CM history |  |  |
| 11.  Christadoulou et al.  2019  South Africa | Home visiting and antenatal depression affect the quality of mother and child interactions in South Africa. ^[[11]](#footnote-11)^ | 1238 healthy pregnant women.  Low income, low education,  Not reported - likely native languages | Single | To examine whether maternal depressed mood at birth moderated the protective effect of a home-visiting intervention on the quality of caregiving for children growing up in a low- and middle-income country. | Peers – ‘mentor mothers’ - Philani Program | Limited details provided | 3 years post-natal | Strong design | Secondary | 5 |
|  |  |  |  |  | Mother/infant dyad |  |  |  |  |  |
|  |  |  | Social Vulnerability |  | Home-based |  |  | Insign. impact |  |  |
| 12.  Cupples et al  2011  UK- Ireland | RCT of peer-mentoring for first-time mothers in socially disadvantaged areas (the MOMENTS Study). ^[[12]](#footnote-12)^ | 343 healthy pregnant women.  Majority low income, aged between 16-30yrs.  English only | Single | To examine the effect of an innovative tailored peer-mentoring programme, based on perceived needs, for first-time mothers in socio-economically deprived communities. | Peer mentors | Limited details provided | 1 year postnatal | Strong design | Secondary | 5 |
|  |  |  |  |  | Pregnant women |  |  |  |  |  |
|  |  |  | Social vulnerability |  | Home visit or phone call |  |  | Insign. impact |  |  |
| 13.  DuMont et al  2008  US | Healthy Families New York (HFNY) randomized trial: effects on early child abuse and neglect. ^[[13]](#footnote-13)^ | 1173 pregnant women  Assessed as at risk for child abuse and neglect, low income, white 34%, African American 45%, Latine 18%, low education, low income.  Spanish or English | Multiple | To evaluate the effects of a home visiting program modelled after Healthy Families America on parenting behaviours in the first 2 years of life. | Para-professionals | Details provided | 2 years postnatal | Mod | Secondary | 8 |
|  |  |  |  |  | Pregnant woman and child |  |  |  |  |  |
|  |  |  | Social vulnerability  At risk child maltreatment |  | Home-based |  |  | Sign. impact on mother |  |  |
|  |  |  |  |  |  |  |  | Sign. impact on child |  |  |
| 14.  Eckenrode et al  2000  US | Preventing child abuse and neglect with a program of nurse home visitation: the limiting effects of domestic violence. ^[[14]](#footnote-14)^ | 400 pregnant women.  Low income, 47% under 19 yrs  Not reported - Likely English only | Multiple | To investigate whether the presence of domestic violence limits the effects of nurse home visitation interventions in reducing substantiated reports of child abuse and neglect. | Healthy Families New York (HFNY)  Nurse | Limited details | 15 years | Mod | Tertiary | 8 |
|  |  |  |  |  | Pregnant women |  |  |  |  |  |
|  |  |  | Social vulnerability  IPV  Child maltreatment |  | Home-based |  |  | Mixed  Some impact mother and child |  |  |
| 15.  Enoch et al  2016  US | A Prospective Cohort Study of Influences on Externalizing Behaviors Across Childhood: Results from a Nurse Home Visiting Randomized Controlled Trial. ^[[15]](#footnote-15)^ | 600 pregnant women  Predominantly African-American  Not reported - Likely English only | Single | To investigate the impact of maternal and genetic influences together with an NHV program, on the development of externalizing behaviours in children aged 2 to 18 years, including the externalizing behaviours of alcohol and drug use disorder (AUD, DUD) and smoking behaviours at age 18 years. | Nurse | Limited details provided | 18 years | Mod | Secondary | 6 |
|  |  |  |  |  | Pregnant women |  |  |  |  |  |
|  |  |  | Social vulnerability |  | Home-based |  |  | Mixed  Some impact mother and child |  |  |
| 16.  Feinberg et al  2008  US | Establishing family foundations: intervention effects on co-parenting, parent/infant well-being, and parent-child relations. ^[[16]](#footnote-16)^ | 169 healthy pregnant women  90% White women, average income.  Not reported - Likely English only | Single | Investigation of whether a brief, universal prevention program is able to enhance parent coordination and mutual support in parenting roles. | Family Foundations (FF) counsellors | Limited details provided | 6 months post intervention | Mod | Primary | 6 |
|  |  |  |  |  | Couples |  |  |  |  |  |
|  |  |  | Prevention |  | Community |  |  | Sign. impact on mother |  |  |
|  |  |  |  |  |  |  |  | Sign. impact on child |  |  |
| 17.  Feinberg et al  2016  US | Preventive Effects on Birth Outcomes: Buffering Impact of Maternal Stress, Depression, and Anxiety. ^[[17]](#footnote-17)^ | 259 pregnant women  Not reported - Likely English only | Single | Examines whether Family Foundations (FF)—a transition to parenthood program for couples focused on promoting co-parenting quality, with previously documented impact on maternal stress, depression, and anxiety—can buffer the negative effects of maternal mental health problems. | Family Foundations (FF) counsellors | Limited details provided | 40 weeks gestation | Strong design | Primary | 7 |
|  |  |  |  |  | Couples |  |  |  |  |  |
|  |  |  | Prevention |  | Community |  |  | Sign. impact on mother |  |  |
|  |  |  |  |  |  |  |  | Sign. impact on child |  |  |
| 18.  Field et al  2004  US | Massage therapy effects on depressed pregnant women. ^[[18]](#footnote-18)^ | 84 depressed pregnant women.  46% Caucasian, 39% Latine, 12% African American, 3% Asia  Not reported - Likely English only | Single | To assess a more cost-effective form of massage therapy, namely having the ‘significant other’ instead of a massage therapist provide the massages. In addition, we assessed the massage therapy effects on fetal activity | Massage by partner | High level of detail provided | 16 weeks (end of intervention) | Mod | Tertiary | 5 |
|  |  |  |  |  | Couples |  |  |  |  |  |
|  |  |  | Perinatal depression |  | Community |  |  | Sign. impact on mother |  |  |
|  |  |  |  |  |  |  |  | Sign. impact on child |  |  |
| 19.  Field  2009  US | Pregnancy massage reduces prematurity, low birthweight and postpartum depression. ^[[19]](#footnote-19)^ | 200 Depressed pregnant women.  57% Latine, 38% African-American, 5% non-Latine, 22% diagnosed with major depressive disorder  Not reported- Likely English only | Single | To assess the effects of pregnancy massage by significant others on prenatal measures as well as perinatal outcomes and in a larger sample. | Massage by partner | High level of detail provided. | Birth of child | Mod | Tertiary | 5 |
|  |  |  |  |  | Couples |  |  |  |  |  |
|  |  |  | Perinatal depression |  | Community |  |  | Sign. impact on mother |  |  |
|  |  |  |  |  |  |  |  | Sign. impact on child |  |  |
| 20.  Field  2012  US | Yoga and massage therapy reduce prenatal depression and prematurity. ^[[20]](#footnote-20)^ | 84 depressed pregnant women  38% Latine, 40% African American, 12% non-Latine white.  Not reported - Likely English only | Single | To compare the effects of massage therapy, yoga postures and standard prenatal care groups of depressed pregnant women from the same clinic population as previous studies. | Yoga instructor and massage therapist | Limited details provided | Post intervention (32 wks gestation) | Mod | Tertiary | 3 |
|  |  |  |  |  | Pregnant women |  |  |  |  |  |
|  |  |  | Perinatal depression |  | Community |  |  | Sign. impact on mother |  |  |
|  |  |  |  |  |  |  |  | Sign. impact on child |  |  |
| 21.  Fischer et al  2006  Vienna | Methadone versus buprenorphine in pregnant addicts: a double-blind, double-dummy comparison study. ^[[21]](#footnote-21)^ | 18 pregnant opioid-dependent women  Average 25 years old  Not reported – likely Viennese German | Single | The aims of this study were to provide a preliminary indication of the relative safety and efficacy of buprenorphine and methadone in opioid-dependent pregnant women | Medical - Medication | High level of detail provided | Birth of child | Mod | Tertiary | 2 |
|  |  |  |  |  | Pregnant woman |  |  |  |  |  |
|  |  |  | Opioid dependent |  | Clinic |  |  | Insign impact on mother or child |  |  |
| 22.  Fisher et al  2016  Australia | Gender-informed, psychoeducational programme for couples to prevent postnatal common mental disorders among primiparous women: cluster randomised controlled trial. ^[[22]](#footnote-22)^ | 400 pregnant women  English only | Single | The aim was to determine whether What Were We Thinking (WWWT) a gender-informed, psychoeducational programme for couples and babies can prevent PCMD (Postpartum Common Mental Health Disorders) among primiparous women 6 months postpartum. | Nurse | High level of detail provided | 6 months postnatal | Strong design | Primary | 8 |
|  |  |  | Prevention |  | Family - Pregnant women partner and child |  |  | Mixed  Some impact mother |  |  |
|  |  |  |  |  | Community |  |  | Insign child |  |  |
| 23.  Fonagy et al  2016  UK | Randomized controlled trial of parent-infant psychotherapy for parents with mental health problems and young infants. ^[[23]](#footnote-23)^ | 76 dyads  37% had higher levels of education, mostly White  Not reported - likely English only | Multiple | This randomized controlled trial investigated the outcomes of PIP for parents with mental health problems who also were experiencing high levels of social adversity and their young infants (<12 months). | PIP psychotherapy therapists | Limited detail provided | 12 months postnatal | Strong design | Tertiary | 6 |
|  |  |  | Perinatal MH  Social vulnerability |  | Family - Parents and infants |  |  | Mixed  Sign. impact on mother |  |  |
|  |  |  |  |  | Clinic |  |  | Insign. Child outcomes |  |  |
| 24.  Fraser et al  2000  Australia | Home visiting intervention for vulnerable families with newborns: follow-up results of a randomized controlled trial. ^[[24]](#footnote-24)^ | 181 healthy pregnant women  Mostly White, with 5.6% Aboriginal or TI  English only | Single | The overall aim of this study was to evaluate the effectiveness of home visiting as a prevention and early intervention strategy using child health nurses, social workers, and parent aides. | Nurse | Limited details provided | 18 months postnatal | Strong design | Secondary | 7 |
|  |  |  |  |  | Pregnant woman and infant/child |  |  |  |  |  |
|  |  |  | Prevention and early intervention |  | Home-based |  |  | Small impact on Mother and child in short term |  |  |
| 25.  Guo et al  2020  China | Preventing Postpartum Depression with Mindful Self-Compassion Intervention: A Randomized Control Study. ^[[25]](#footnote-25)^ | 354 pregnant women with depression or anxiety symptoms  Chinese only | Single | To evaluate the effectiveness of a Web-based, antenatal psychological intervention for preventing the development of PPD among women with high risk.  Based on MBSP (mindful self-compassion program) | Mindfulness programme | Few details provided | 12 months postnatal | Strong design | Secondary | 5 |
|  |  |  |  |  | Pregnant woman |  |  |  |  |  |
|  |  |  | Perinatal depression or anxiety |  | Virtual - Can be home - Internet |  |  | Sign. impact on mother |  |  |
|  |  |  |  |  |  |  |  | Sign. impact on child |  |  |
| 26.  Gureje et al  2019  Nigeria | High- versus low-intensity interventions for perinatal depression delivered by non-specialist primary maternal care providers in Nigeria: cluster randomised controlled trial (the EXPONATE trial). ^[[26]](#footnote-26)^ | 686 pregnant women  Low education levels, low income  Yoruba only | Single | To compare high-intensity treatment (HIT) with low-intensity treatment (LIT) for perinatal depression | MH intervention  By Midwives (Primary Maternal Care Provider - PMCPs) | Moderate level of detail provided | 12 months postnatal | Strong design | Tertiary | 4 |
|  |  |  |  |  | Pregnant women |  |  |  |  |  |
|  |  |  | Perinatal depression |  | Clinic |  |  | Some impact |  |  |
| 27.  Gutterman, NB et al.  2023  US | Engaging fathers to strengthen the impact of early home visitation on physical child abuse risk: Findings from the dads matter-HV (DM-HV) randomised controlled trial.^[[27]](#footnote-27)^ | 204 families  Intervention (103 families): 61% Latinx, 31% African American, 8% Other  English and Spanish | Multiple | This study examines the effectiveness of Dads Matter-HV (“DM-HV”), a father-inclusion  enhancement to home visitation, and hypothesized mediators of impact. | Professionals or trained paraprofessionals | Moderate level of detail provided | 12 month | Strong design | Secondary | 9 |
|  |  |  |  |  | Fathers and mothers |  |  | Sign. impact |  |  |
|  |  |  | Prevention  Social vulnerability  Eligible for Home visiting programme |  | Home-based |  |  | Insign. when services initiated prenatally |  |  |
| 28.  Huang  2021  China | Effects of Internet-Based Support Program on Parenting Outcomes for Primiparous Women: A Pilot Study. ^[[28]](#footnote-28)^ | 44 healthy pregnant women  Chinese only | Single | To pilot the internet-based support program (ISP) was designed to improve the Chinese primiparous women’s parenting ability, mental wellbeing, and social support. | Parenting programme | Moderate level of detail provided | 3 months post intervention | Strong design | Primary | 5 |
|  |  |  |  |  | Pregnant women |  |  |  |  |  |
|  |  |  | Prevention |  | Virtual - Internet – can be home |  |  | Mixed  Sign. impact on mother |  |  |
|  |  |  |  |  |  |  |  | Insign. Child outcomes |  |  |
| 29.  Johnston et al  2006  US | Healthy steps in an integrated delivery system: child and parent outcomes at 30 months. ^[[29]](#footnote-29)^ | 439 healthy pregnant women  English only | Single | To test the effects of the Healthy Steps for Young Children program (HS) (which supports parents managing children's developmental and behavioural issues)—with and without a prenatal component—on child health and development, parenting practices, and parental well-being. | Nurse, social worker and MH clinician | High level of detail provided | 30 months postnatal | Mod | Primary | 8 |
|  |  |  |  |  | Pregnant women |  |  |  |  |  |
|  |  |  | Prevention |  | Home-based |  |  | Sign. impact on mother |  |  |
|  |  |  |  |  |  |  |  | Sign. impact on child |  |  |
| 30.  Kamalifard et al  2013  Iran | The effect of peers support on postpartum depression: a single-blind randomized clinical trial. ^[[30]](#footnote-30)^ | 100 healthy pregnant women  66% high school or less  Persian only | Single | This study aimed to investigate the effect of mothers receiving peer support on postpartum depression. | Peer support | Limited details | 8 weeks post- partum | Strong design | Primary | 3 |
|  |  |  |  |  | Pregnant women |  |  |  |  |  |
|  |  |  | Prevention PPD |  | Virtual - Can be home - Telephone |  |  | Sign. impact on mother  Sign. impact on child |  |  |
| 31.  Kenyon et al  2016  UK | Lay support for pregnant women with social risk: a randomised controlled trial. ^[[31]](#footnote-31)^ | 1324 pregnant women  53% European, 48% British, 4% eastern European, 1% other, low income  Not reported – Likely English only | Single | We sought evidence of effectiveness of lay support to improve maternal and child outcomes in disadvantaged families. | Lay support | Limited details of intervention provided | 8-12 weeks post-partum | Strong design | Secondary | 7 |
|  |  |  |  |  | Pregnant women |  |  |  |  |  |
|  |  |  | Social vulnerability |  | Clinic and some home-based |  |  | Sign. impact on Mother |  |  |
|  |  |  |  |  |  |  |  | Sign. impact on child |  |  |
| 32.  Kersten-Alverez et  2010  Netherlands | Long-term effects of a home-visiting intervention for depressed mothers and their infants. ^[[32]](#footnote-32)^ | 95 depressed pregnant women  Not reported - Likely Dutch only | Single | To examine the longer-term effects of an intervention for mothers with postpartum depression and their infants at school-age. | MH clinician | Limited details provided | 68 months postnatal | Strong design | Tertiary | 5 |
|  |  |  |  |  | Mother/baby dyad |  |  |  |  |  |
|  |  |  | Perinatal depression |  | Home-based |  |  | Insign. impact |  |  |
| 33.  Kiely et al  2010  US | An integrated intervention to reduce intimate partner violence in pregnancy: a randomized controlled trial. ^[[33]](#footnote-33)^ | 1044 pregnant women with IPV  100% African American women, low income  English only | Single | To estimate the efficacy of a cognitive behavioural intervention administered as part of a randomized controlled trial (RCT) during prenatal care (PNC) in reducing IPV recurrence during pregnancy and improving birth outcomes (LBW and PTB) in a population of African-American residents of Washington, DC | Psycho-behavioural intervention MH clinician | Moderate details provided | 34-38 weeks gestation | Strong design | Tertiary | 4 |
|  |  |  |  |  | Pregnant women |  |  |  |  |  |
|  |  |  | IPV |  | Clinic |  |  | Sign. impact on mother |  |  |
|  |  |  |  |  |  |  |  | Sign. impact on child |  |  |
| 34.  Leng,L et al  2023  China | Antenatal mobile-delivered mindfulness-based intervention to reduce perinatal depression risk and improve obstetric and neonatal outcomes: A randomized controlled trial.^[[34]](#footnote-34)^ | 75 adult pregnant women who exhibited elevated levels of stress  100% Chinese, 63% middle household income  Chinese only | Single | The present study aimed to investigate the effect of a mobile-delivered therapist's guided four-immeasurable MBI - “Thriving in Pregnancy - Cultivating the Four Immeasurables” i.e., loving-kindness, compassion, appreciative joy, and equanimity meditations. (Thrive-Pregnancy) compared to a web-based perinatal education program on PND. | App-based  Psychotherapists | Moderate details provided | 12-28 weeks gestation | Strong design | Secondary | 6 |
|  |  |  |  |  | Mothers |  |  | Sign. Impact on mother |  |  |
|  |  |  | Elevated levels of stress living in community |  | Virtual - On-line |  |  | Sign. Impact on child |  |  |
| 35.  Maimburg, R. D. and M. Vaeth  2015  Denmark | Postpartum depression among first-time mothers - results from a parallel randomised trial.^[[35]](#footnote-35)^ | 1193 pregnant women (603 in intervention)  99% in relationship with partner  Danish only | Single | To compare the risk of [postpartum depression](https://www.sciencedirect.com/topics/medicine-and-dentistry/postpartum-depression) among [nulliparous](https://www.sciencedirect.com/topics/medicine-and-dentistry/nulliparous) women enrolled in a structured antenatal programme, with nulliparous women allocated to standard care as well as to identify obstetric characteristics in women at risk of developing postpartum depression. | Midwife delivered Structured antenatal education programme | Some detail provided and reference where to find out more | 6 weeks post delivery | Strong design | Primary | 4 |
|  |  |  |  |  | Pregnant women, fathers were invited |  |  | Insign. impact |  |  |
|  |  |  | Prevent PPD |  | Group community class |  |  |  |  |  |
| 36.  Makrides, M., et al.  2010  Australia | Effect of DHA supplementation during pregnancy on maternal depression and neurodevelopment of young children: A randomized controlled trial. ^[[36]](#footnote-36)^ | 2399 pregnant women  <21 weeks gestation  English only | Single | Test whether DHA supplementation during last half of pregnancy reduced risk of depressed mood post-partum and improved early cognition in offspring. | DHA Supplement | High level detail provided | 6 months for mother's outcomes  18 months for child outcomes | Strong design | Primary | 2 |
|  |  |  |  |  | Pregnant women |  |  |  |  |  |
|  |  |  | Prevent PPD |  | Clinic |  |  | Insign. impact |  |  |
| 37.  (linked to [#48])  Maselko, J, et al  2015  Pakistan | Effect of an early perinatal depression intervention on long-term child development outcomes: follow-up of the Thinking Healthy Programme randomised controlled trial. ^[[37]](#footnote-37)^ | 584 pregnant women  Not reported - Likely local languages | Multiple | We aimed to assess the effect of this same intervention from 2005 [#48 in this table] on the cognitive, socioemotional, and physical development of children at around age 7 years. | community workers | Moderate level of detail provided | 7 years post intervention | Strong design | Tertiary | 6 |
|  |  |  |  |  | Woman and child |  |  |  |  |  |
|  |  |  | Post-natal depression (PPD)  Social vulnerability |  | Home-based |  |  | Insign. impact |  |  |
| 38.  McConnell, M et al.  2022  US | Effect of an Intensive Nurse Home Visiting Program on Adverse Birth Outcomes in a Medicaid-Eligible Population: A Randomized Clinical Trial.^[[38]](#footnote-38)^ | 5670 pregnant women, 3806 in programme, 1864 in control.  Low income  < 28 weeks gestation  Spanish or English | single | To determine the effect of an intensive nurse home visiting program on a composite outcome of any of preterm birth, low birth weight, small for gestational age, or perinatal mortality. | Nurses | Limited detail provided | 2 years postnatal | Strong design | Secondary | 8 |
|  |  |  |  |  | Mother and child |  |  | Insign. Mother or child |  |  |
|  |  |  | Low Income, Medicaid |  | Home  Some telehealth visits (phone or video) |  |  |  |  |  |
| 39.  McFarlane, E., et al.  2013  Hawaii, US | Maternal relationship security as a moderator of home visiting impacts on maternal psychosocial functioning. ^[[39]](#footnote-39)^ | 643 pregnant women  64% below poverty line, 30% adolescents, approx. 32% Native Hawaiian or Pacific Islander, 27% Asian or Filipino, 13% Caucasian, 28% Other  English only | Multiple | The present analyses’ primary objectives were to build on our earlier reports by assessing overall Hawaii’s Healthy Start Program (HSP) impacts on maternal psychosocial functioning when children were 7 to 9 years old and by testing maternal attributes, including maternal relationship security, as moderators of short- and longer-term program impacts. | Para-professionals | Limited detail provided however reference where to find more | 7&9 years | Strong design | Secondary | 7 |
|  |  |  |  |  | Mother and child |  |  |  |  |  |
|  |  |  | Social vulnerability  Assessed as at risk for child maltreatment |  | Home-based |  |  | Insign. Mother or child |  |  |
| 40.  Milgrom J, et al.  2015  Australia | Feasibility study and pilot randomised trial of an antenatal depression treatment with infant follow-up. ^[[40]](#footnote-40)^ | 54 pregnant women with perinatal depression diagnosis  English only | Single | Assessed the feasibility of running an RCT and also provided preliminary data on the efficacy of the new CBT programme, compared to usual care, in a sample of pregnant women with a diagnosed depressive disorder. | MH Clinician - psychologists | Limited detail provided | 9 months postnatal | Strong design | tertiary | 6 |
|  |  |  |  |  | couples |  |  |  |  |  |
|  |  |  | Peri-natal depression |  | Clinic setting |  |  | Sign. impact on mother |  |  |
|  |  |  |  |  |  |  |  | Sign. impact on child |  |  |
| 41.  Mohd Shukri NH, et al.  2019  Malaysia | Randomized controlled trial investigating the effects of a breastfeeding relaxation intervention on maternal psychological state, breast milk outcomes, and infant behavior and growth. ^[[41]](#footnote-41)^ | 64 pregnant women  First time mothers, primarily low income and are exclusively breastfeeding  Not reported - Likely Malay only | Single | MOM study - To investigate physiological and psychological aspects of mother–infant signalling during breastfeeding experimentally, testing the effects of an audio relaxation intervention on maternal psychological state, breast milk intake, milk cortisol levels, and infant behavior and growth. | Audio | Moderate level detail provided | 14-18 weeks post-partum | Strong design | Secondary | 3 |
|  |  |  |  |  | Pregnant woman and infant dyad |  |  |  |  |  |
|  |  |  | Social vulnerability |  | Home-based |  |  | Mixed  Some impact at early timepoint |  |  |
|  |  |  |  |  |  |  |  | Insign. later timepoints |  |  |
| 42.  Nicholson, s et al.  2022  Australia | Supporting early infant relationships and reducing maternal distress with the Newborn Behavioral Observations: A randomized controlled effectiveness trial.^[[42]](#footnote-42)^ | 111 pregnant women  <36 weeks gestation, aged 20 yrs or over.  Caucasian - 62%  Asian - 11.7%  Middle Eastern - 2.7%  Muslim - 2.7%  English speaking | Multiple | The study examined the effectiveness of the NBO in a population with antenatal distress and risk of postnatal depression (PND). | NBO accredited midwife or MCH nurse | Moderate level detail provided | 4 months postnatal | Strong | Secondary | 6 |
|  |  |  |  |  | Pregnant woman and infant dyad |  |  | Some impact mother. |  |  |
|  |  |  | Risk of depression, anxiety and psychosocial factors |  | Home-based |  |  | Some impact child |  |  |
| 43.  Olds DL, et al.  2002  US | Home Visiting by Paraprofessionals and by Nurses: A Randomized, Controlled Trial. ^[[43]](#footnote-43)^ | 1178 pregnant women  50% Latine, 36% Caucasian. Primarily low income, qualify for Medicaid or have no insurance, low education, and average 19 years old.  English or Spanish | Single | Examine the effectiveness of home visiting by paraprofessionals and by nurses as separate means of improving maternal and child health when both types of visitors are trained in a program model that has demonstrated effectiveness when delivered by nurses. | Para-professionals and Nurses | Limited level of detail provided | 24 months postnatal | Strong design | Secondary | 8 |
|  |  |  |  |  | Pregnant woman and child |  |  |  |  |  |
|  |  |  | Social vulnerability |  | Home-based |  |  | Mixed  Small impact para-professionals |  |  |
|  |  |  |  |  |  |  |  | Sign. impact mother and child with nurses |  |  |
| 44.  Olds, D. L., et al.  2004  US | Effects of home visits by paraprofessionals and by nurses: Age 4 follow-up results of a randomized trial. ^[[44]](#footnote-44)^ | As per [#43 above] |  | To examine the effects of prenatal and infancy home visiting by paraprofessionals and by nurses from child age 2 through age 4 years. |  | Moderate level of detail provided | 4 years old follow up of study | Strong design | Secondary | 8 |
|  |  |  |  |  |  |  |  | Sign. impact para-professionals mother and child |  |  |
|  |  |  |  |  |  |  |  | Sign. impact nurses, mother and child |  |  |
| 45.  Olds, D. L., et al  2007  US | Effects of nurse home visiting on maternal and child functioning: Age-9 follow-up of a randomized trial. ^[[45]](#footnote-45)^ | 1167 pregnant women  Primarily African American 92%, 98% unmarried, 64% 18 years or younger, 85% households w incomes <FPL (Family Poverty Line)  Not reported – Likely English only | Single | To test the effect of prenatal and infancy home visits by nurses on mothers’ fertility and children’s functioning 7 years after the program ended at child age 2. | Nurses | Limited level of detail provided | 9 years old | Strong design | Secondary | 8 |
|  |  |  |  |  | Pregnant women and child |  |  |  |  |  |
|  |  |  | Social vulnerability |  | Home-based |  |  | Sign. Impact mother |  |  |
|  |  |  |  |  |  |  |  | Sign. Impact child |  |  |
| 46.  Olds, D. L., et al.  2010  US | Enduring effects of prenatal and infancy home visiting by nurses on maternal life course and government spending: Follow-up of a randomized trial among children at age 12 years. ^[[46]](#footnote-46)^ | As per [#41 above] | Single | To test the effects of prenatal and infancy home visiting by nurses on mothers' fertility, partner relationships, and economic self-sufficiency and on government spending through age 12 years of their firstborn child.  [linked to #42] | Nurses | Moderate level of detail provided | 12 years old | Strong design | Secondary | 8 |
|  |  |  |  |  | Pregnant women and child |  |  |  |  |  |
|  |  |  | Social vulnerability |  | Home-based |  |  | Mixed  Sign. Impact mother |  |  |
|  |  |  |  |  |  |  |  | Insign. Impact child |  |  |
| 47.  Onozawa, K. et al.  2001  London, UK | Infant massage improves mother-infant interaction for mothers with postnatal depression. ^[[47]](#footnote-47)^ | 34 pregnant women with postnatal depression  75% class spoke English | Single | To test the effect of infant massage by mothers with postnatal depression on mother–infant interaction. | Massage therapists | High level detail provided | Post 5-week intervention | Moderate design | Tertiary | 4 |
|  |  |  |  |  | Pregnant woman and infant |  |  |  |  |  |
|  |  |  | Post-natal depression |  | Clinic - group |  |  | Sign. Impact mother |  |  |
|  |  |  |  |  |  |  |  | Sign. Impact child |  |  |
| 48. (linked to [#37])  Rahman, A., et al.  2008  Pakistan | Cognitive behaviour therapy-based intervention by community health workers for mothers with depression and their infants in rural Pakistan: A cluster-randomised controlled trial. ^[[48]](#footnote-48)^ | 903 pregnant women  51% women were financially empowered, 21% from very poor neighbourhoods, all lived in a rural setting.  Not reported - Likely local languages | Multiple | To test the effectiveness of an integrated cognitive behaviour therapy-based intervention into the routine work of community-based primary health workers (called lady health Workers) in rural Pakistan and assessed the effect of this intervention on maternal depression and infant outcomes.  [linked to #37] | Para-professionals | Moderate level of detail provided | 1 year | Strong design | Tertiary | 6 |
|  |  |  |  |  | Pregnant women |  |  |  |  |  |
|  |  |  | Post-natal depression (PPD)  Social vulnerability |  | Home-based |  |  | Sign. Impact mother |  |  |
|  |  |  |  |  |  |  |  | Sign. Impact child |  |  |
| 49.  Robling, F.V., et al.  2022  England | Nurse-led home-visitation programme for first-time mothers in reducing maltreatment and improving child health and development (BB:2-6): longer-term outcomes from a randomised cohort using data linkage.^[[49]](#footnote-49)^ | 1618 pregnant women  19 years or less, <25 weeks gestation  English | Single | This study followed a cohort of mothers and children who had participated in the BB:0–2 trial for a further 5 years using administrative data only. To determine the effectiveness of the Family Nurse Partnership (FNP) programme when added to usually provided health and social care in reducing maltreatment, when compared with usually provided health and social care alone. Secondary: to determine programme effectiveness in reducing maltreatment, medium-term programme outcomes such as subsequent pregnancies, school readiness and educational outcomes, the impact of moderators of programme effect and the cost and consequences of the programme. | Trained Family Nurses | Limited detail provided | Children were 7 years old. | Strong | Primary | 7 |
|  |  |  |  |  | Pregnant women |  |  | Insign. Impact mother |  |  |
|  |  |  | Young parents, social vulnerability |  | Home based |  |  | Small impact child |  |  |
| 50.  Rotheram-Borus, M. J., et al.  2019  South Africa | The association of maternal alcohol use and paraprofessional home visiting with children’s health: A randomized controlled trial. ^[[50]](#footnote-50)^ | 1236 pregnant women  47% low income, 19% employed, 31% lived in formal housing  Not reported – likely local languages | Multiple | This study examines the effect of a home visiting intervention on maternal alcohol use, problematic drinking, and the association of home visiting and alcohol use on children’s behavioral, cognitive, and health outcomes at five time points over 5 years. | Para-professionals | Moderate level detail provided | 60 months postnatal | Moderate | Secondary | 6 |
|  |  |  |  |  | Pregnant woman and child |  |  |  |  |  |
|  |  |  | Social vulnerability |  | Community-based teams - Clinic and Home |  |  | Sign. Impact on mother |  |  |
|  |  |  |  |  |  |  |  | Some impact child |  |  |
| 51.  Tomlinson, M., et al.  2015  South Africa | Community health workers can improve child growth of antenatally-depressed, South African mothers: a cluster randomized controlled trial. ^[[51]](#footnote-51)^ | 1238 women  Antenatally depressed women who are pregnant or with infant up to 6 months age  Not reported - likely local languages | Multiple | To assess if home visits by community health workers (CHW) can improve growth outcomes for children of mothers who are antenatally depressed. | Women identified by community | Moderate level detail provided | 6 months post- partum | Strong design | Tertiary | 9 |
|  |  |  |  |  | Mothers and infants |  |  |  |  |  |
|  |  |  | Perinatal depression  Social vulnerability |  | Home-based |  |  | Insign. Impact mother |  |  |
|  |  |  |  |  |  |  |  | Sign. Impact on child |  |  |
| 52.  Tripathy, P., et al.  2010  India | Effect of a participatory intervention with women's groups on birth outcomes and maternal depression in Jharkhand and Orissa, India: A cluster-randomised controlled trial. ^[[52]](#footnote-52)^ | 4692 births  Recorded 111,006 group attendances over 3 years. 74,715 (67%) of these were from married women of reproductive age, 15,030 (14%) from adolescent girls, 10,452 (9%) from men, and 10,809 (10%) from elderly women.  Majority low-income rural communities  Ability to speak a local language | Multiple | We hypothesised that a participatory intervention with women's groups could reduce neonatal mortality by at least 25% in underserved tribal communities of eastern India, and improve home-care practices and health-seeking behaviour of pregnant and postnatal women, and their family members; and that the women's group intervention could reduce maternal depression in the intervention areas by 30%. | Volunteers | High level detail provided | 3 years | Strong design | Primary | 8 |
|  |  |  |  |  | Women |  |  |  |  |  |
|  |  |  | Social vulnerability  Live in Rural setting |  | Community |  |  | Insign. Impact mother |  |  |
|  |  |  |  |  |  |  |  | Sign. Impact child |  |  |
| 53.  Urizar, G. G., Jr.  2011  US | Impact of a prenatal cognitive-behavioural stress management intervention on salivary cortisol levels in low-income mothers and their infants. ^[[53]](#footnote-53)^ | 86 pregnant women  75% born Mexico/central America, 6% African American, 2% Caucasian  English and Spanish | Multiple | To test whether a prenatal cognitive behavioural stress management (CBSM) intervention would be effective in regulating salivary cortisol (a biological marker of stress) and self-reported stress levels among mothers and their infants at six and 18 months postpartum, relative to two control groups. | Psychologists | High level detail provided | 18 months | Moderate design | Secondary | 6 |
|  |  |  |  |  | Women |  |  |  |  |  |
|  |  |  | Social vulnerability  Assessed as high-risk child maltreatment  Perinatal Depression, anxiety or stress. |  | Group clinic based |  |  | Sign. Impact mother |  |  |
|  |  |  |  |  |  |  |  | Sign. Impact child |  |  |
| 54.  Van Doesum KT et al.  2008  Netherlands | A randomized controlled trial of a home-visiting intervention aimed at preventing relationship problems in depressed mothers and their infants. ^[[54]](#footnote-54)^ | 85 pregnant women  Women with perinatal depression  Dutch language | Single | To test the effect of a mother-baby intervention on the quality of mother-child interaction, infant-mother attachment security, and infant socioemotional functioning in a group of depressed mothers with infants aged 1-12 months. | Prevention specialists – trained post grad | High level detail provided | 6 months | Strong design | Tertiary | 6 |
|  |  |  |  |  | Woman and infant dyad |  |  |  |  |  |
|  |  |  | Perinatal depression |  | Home-based |  |  | Sign. Impact mother |  |  |
|  |  |  |  |  |  |  |  | Sign. Impact child |  |  |
| 55.  Walkup, J. T., et al  2009  US | Randomized controlled trial of paraprofessional-delivered in-home intervention for young reservation-based American Indian mothers.^[[55]](#footnote-55)^ | 167 pregnant women  Young, reservation-based American Indian (AI) mothers  English and native languages | Multiple | To evaluate the efficacy of a paraprofessional-delivered, home-visiting intervention among young, reservation-based American Indian (AI) mothers on parenting knowledge, involvement, and maternal and infant outcomes. | Para-professionals | High level detail provided | 12 months | Strong design | Secondary | 10 |
|  |  |  |  |  | Woman and infant dyad |  |  |  |  |  |
|  |  |  | Adolescents  Live in Rural setting |  | Home-based |  |  | Sign. Impact mother |  |  |
|  |  |  |  |  |  |  |  | Sign. Impact child |  |  |
| 56.  Werner, E. A., et al.  2016  US | PREPP: Postpartum depression prevention through the mother–infant dyad. ^[[56]](#footnote-56)^ | 54 pregnant women  57% Latine, 38.5% non-Latine, African American 15.4%, Caucasian 15.4%, Asian 7%, Biracial 3.8% and other  English only | single | The goal of the current study was to examine the effectiveness of Practical Resources for Effective Postpartum Parenting (PREPP). PREPP is a new PPD prevention protocol that aims to treat women at risk for PPD by promoting maternally–mediated behavioural changes in their infants, while also including mother–focused skills. | Psychologist | Moderate level detail provided | 10 weeks post- partum | Moderate design | Secondary | 6 |
|  |  |  |  |  | Woman and infant dyad |  |  |  |  |  |
|  |  |  | Prevention PPD |  | Home-based |  |  | Sign. Impact mother |  |  |
|  |  |  |  |  |  |  |  | Sign. Impact child |  |  |
| 57.  Zielinski, D. S., et al.  2009  US | Nurse home visitation and the prevention of child maltreatment: impact on the timing of official reports. ^[[57]](#footnote-57)^ | 400 low-income pregnant women in the original RCT, 324 completed assessments at 15 years.  89% Caucasian, 11% African American  Likely English only | Multiple | In this paper they examine the temporal features of maltreatment that were affected by the Nurse Family Partnership (NFP). | Nurses | Moderate level detail provided | 15 years | Medium design | Secondary | 8 |
|  |  |  |  |  | Pregnant women |  |  |  |  |  |
|  |  |  | Social vulnerability  And or  Adolescent |  | Home-based |  |  | Nil mother |  |  |
|  |  |  |  |  |  |  |  | Sign. Impact child |  |  |

1. Armstrong KL, Fraser JA, Dadds MR, Morris J. Promoting secure attachment, maternal mood and child health in a vulnerable population: a randomized controlled trial. J Paediatr Child Health. 2000 Dec;36(6):555-62. doi: 10.1046/j.1440-1754.2000.00591.x. PMID: 11115031. [↑](#footnote-ref-1)
2. Austin MP, Frilingos M, Lumley J, Hadzi-Pavlovic D, Roncolato W, Acland S, Saint K, Segal N, Parker G. Brief antenatal cognitive behaviour therapy group intervention for the prevention of postnatal depression and anxiety: a randomised controlled trial. J Affect Disord. 2008 Jan;105(1-3):35-44. doi: 10.1016/j.jad.2007.04.001. Epub 2007 May 8. PMID: 17490753. [↑](#footnote-ref-2)
3. Barlow J, Davis H, McIntosh E, Jarrett P, Mockford C, Stewart-Brown S. Role of home visiting in improving parenting and health in families at risk of abuse and neglect: results of a multicentre randomised controlled trial and economic evaluation. Arch Dis Child. 2007 Mar;92(3):229-33. doi: 10.1136/adc.2006.095117. Epub 2006 Oct 26. PMID: 17068074; PMCID: PMC2083433. [↑](#footnote-ref-3)
4. Barlow A, Mullany B, Neault N, Compton S, Carter A, Hastings R, Billy T, Coho-Mescal V, Lorenzo S, Walkup JT. Effect of a paraprofessional home-visiting intervention on American Indian teen mothers’ and infants’ behavioral risks: a randomized controlled trial. Am J Psychiatry. 2013 Jan;170(1):83-93. doi: 10.1176/appi.ajp.2012.12010121. PMID: 23409290; PMCID: PMC4542092. [↑](#footnote-ref-4)
5. Barlow A, Mullany B, Neault N, Goklish N, Billy T, Hastings R, Lorenzo S, Kee C, Lake K, Redmond C, Carter A, Walkup JT. Paraprofessional-delivered home-visiting intervention for American Indian teen mothers and children: 3-year outcomes from a randomized controlled trial. Am J Psychiatry. 2015 Feb 1;172(2):154-62. doi: 10.1176/appi.ajp.2014.14030332. Epub 2014 Oct 31. PMID: 25321149. [↑](#footnote-ref-5)
6. Barnes J, Senior R, MacPherson K. The utility of volunteer home-visiting support to prevent maternal depression in the first year of life. Child Care Health Dev. 2009 Nov;35(6):807-16. doi: 10.1111/j.1365-2214.2009.01007.x. Epub 2009 Aug 24. PMID: 19719770. [↑](#footnote-ref-6)
7. Barnes J, Stuart J, Allen E, Petrou S, Sturgess J, Barlow J, Macdonald G, Spiby H, Aistrop D, Melhuish E, Kim SW, Elbourne D. Randomized controlled trial and economic evaluation of nurse-led group support for young mothers during pregnancy and the first year postpartum versus usual care. Trials. 2017 Nov 1;18(1):508. doi: 10.1186/s13063-017-2259-y. PMID: 29092713; PMCID: PMC5667036. [↑](#footnote-ref-7)
8. Bartu A, Sharp J, Ludlow J, Doherty DA. Postnatal home visiting for illicit drug-using mothers and their infants: a randomised controlled trial. Aust N Z J Obstet Gynaecol. 2006 Oct;46(5):419-26. doi: 10.1111/j.1479-828X.2006.00628.x. PMID: 16953857. [↑](#footnote-ref-8)
9. Baumgartner JN, Ali M, Gallis JA, Lillie M, Owusu R, Abubakr-Bibilazu S, Adam H, Aborigo R, McEwan E, Zhou Y, Kim ET, Mackness J, Williams JKA, Hembling J. Effect of a lay counselor-delivered integrated maternal mental health and early childhood development group-based intervention in Northern Ghana: a cluster-randomized controlled trial. Glob Ment Health (Camb). 2021 May 26;8:e18. doi: 10.1017/gmh.2021.15. PMID: 34104458; PMCID: PMC8157813. [↑](#footnote-ref-9)
10. Berry OO, Babineau V, Lee S, Feng T, Scorza P, Werner EA, Monk C. Perinatal depression prevention through the mother-infant dyad: The role of maternal childhood maltreatment. J Affect Disord. 2021 Jul 1;290:188-196. doi: 10.1016/j.jad.2021.04.068. Epub 2021 May 2. PMID: 34004400; PMCID: PMC8217280. [↑](#footnote-ref-10)
11. Christodoulou J, Rotheram-Borus MJ, Bradley AK, Tomlinson M. Home Visiting and Antenatal Depression Affect the Quality of Mother and Child Interactions in South Africa. J Am Acad Child Adolesc Psychiatry. 2019 Dec;58(12):1165-1174. doi: 10.1016/j.jaac.2019.03.016. Epub 2019 Mar 26. PMID: 30926571; PMCID: PMC7316421. [↑](#footnote-ref-11)
12. Cupples ME, Stewart MC, Percy A, Hepper P, Murphy C, Halliday HL. A RCT of peer-mentoring for first-time mothers in socially disadvantaged areas (the MOMENTS Study). Arch Dis Child. 2011 Mar;96(3):252-8. doi: 10.1136/adc.2009.167387. Epub 2010 Jun 3. PMID: 20522466. [↑](#footnote-ref-12)
13. DuMont K, Mitchell-Herzfeld S, Greene R, Lee E, Lowenfels A, Rodriguez M, Dorabawila V. Healthy Families New York (HFNY) randomized trial: effects on early child abuse and neglect. Child Abuse Negl. 2008 Mar;32(3):295-315. doi: 10.1016/j.chiabu.2007.07.007. PMID: 18377991. [↑](#footnote-ref-13)
14. Eckenrode J, Ganzel B, Henderson CR Jr, Smith E, Olds DL, Powers J, Cole R, Kitzman H, Sidora K. Preventing child abuse and neglect with a program of nurse home visitation: the limiting effects of domestic violence. JAMA. 2000 Sep 20;284(11):1385-91. doi: 10.1001/jama.284.11.1385. PMID: 10989400. [↑](#footnote-ref-14)
15. Enoch MA, Kitzman H, Smith JA, Anson E, Hodgkinson CA, Goldman D, Olds DL. A Prospective Cohort Study of Influences on Externalizing Behaviors Across Childhood: Results From a Nurse Home Visiting Randomized Controlled Trial. J Am Acad Child Adolesc Psychiatry. 2016 May;55(5):376-82. doi: 10.1016/j.jaac.2016.02.007. Epub 2016 Mar 2. PMID: 27126851; PMCID: PMC4851736. [↑](#footnote-ref-15)
16. Feinberg ME, Kan ML. Establishing family foundations: intervention effects on coparenting, parent/infant well-being, and parent-child relations. J Fam Psychol. 2008 Apr;22(2):253-63. doi: 10.1037/0893-3200.22.2.253. PMID: 18410212; PMCID: PMC3178882. [↑](#footnote-ref-16)
17. Feinberg ME, Jones DE, Roettger ME, Hostetler ML, Sakuma KL, Paul IM, Ehrenthal DB. Preventive Effects on Birth Outcomes: Buffering Impact of Maternal Stress, Depression, and Anxiety. Matern Child Health J. 2016 Jan;20(1):56-65. doi: 10.1007/s10995-015-1801-3. PMID: 26194453. [↑](#footnote-ref-17)
18. Field T, Diego MA, Hernandez-Reif M, Schanberg S, Kuhn C. Massage therapy effects on depressed pregnant women. J Psychosom Obstet Gynaecol. 2004 Jun;25(2):115-22. doi: 10.1080/01674820412331282231. PMID: 15715034. [↑](#footnote-ref-18)
19. Field T, Diego M, Hernandez-Reif M, Deeds O, Figueiredo B. Pregnancy massage reduces prematurity, low birthweight and postpartum depression. Infant Behav Dev. 2009 Dec;32(4):454-60. doi: 10.1016/j.infbeh.2009.07.001. Epub 2009 Jul 30. PMID: 19646762. [↑](#footnote-ref-19)
20. Field T, Diego M, Hernandez-Reif M, Medina L, Delgado J, Hernandez A. Yoga and massage therapy reduce prenatal depression and prematurity. J Bodyw Mov Ther. 2012 Apr;16(2):204-9. doi: 10.1016/j.jbmt.2011.08.002. Epub 2011 Sep 23. PMID: 22464118; PMCID: PMC3319349. [↑](#footnote-ref-20)
21. Fischer G, Ortner R, Rohrmeister K, Jagsch R, Baewert A, Langer M, Aschauer H. Methadone versus buprenorphine in pregnant addicts: a double-blind, double-dummy comparison study. Addiction. 2006 Feb;101(2):275-81. doi: 10.1111/j.1360-0443.2006.01321.x. PMID: 16445556. [↑](#footnote-ref-21)
22. Fisher J, Rowe H, Wynter K, Tran T, Lorgelly P, Amir LH, Proimos J, Ranasinha S, Hiscock H, Bayer J, Cann W. Gender-informed, psychoeducational programme for couples to prevent postnatal common mental disorders among primiparous women: cluster randomised controlled trial. BMJ Open. 2016 Mar 7;6(3):e009396. doi: 10.1136/bmjopen-2015-009396. PMID: 26951210; PMCID: PMC4785308. [↑](#footnote-ref-22)
23. Fonagy P, Sleed M, Baradon T. RANDOMIZED CONTROLLED TRIAL OF PARENT-INFANT PSYCHOTHERAPY FOR PARENTS WITH MENTAL HEALTH PROBLEMS AND YOUNG INFANTS. Infant Ment Health J. 2016 Mar-Apr;37(2):97-114. doi: 10.1002/imhj.21553. Epub 2016 Mar 4. PMID: 26939716. [↑](#footnote-ref-23)
24. Fraser JA, Armstrong KL, Morris JP, Dadds MR. Home visiting intervention for vulnerable families with newborns: follow-up results of a randomized controlled trial. Child Abuse Negl. 2000 Nov;24(11):1399-429. doi: 10.1016/s0145-2134(00)00193-9. PMID: 11128173. [↑](#footnote-ref-24)
25. Guo L, Zhang J, Mu L, Ye Z. Preventing Postpartum Depression With Mindful Self-Compassion Intervention: A Randomized Control Study. J Nerv Ment Dis. 2020 Feb;208(2):101-107. doi: 10.1097/NMD.0000000000001096. PMID: 31868776. [↑](#footnote-ref-25)
26. Gureje O, Oladeji BD, Montgomery AA, Araya R, Bello T, Chisholm D, Groleau D, Kirmayer LJ, Kola L, Olley LB, Tan W, Zelkowitz P. High- versus low-intensity interventions for perinatal depression delivered by non-specialist primary maternal care providers in Nigeria: cluster randomised controlled trial (the EXPONATE trial). Br J Psychiatry. 2019 Sep;215(3):528-535. doi: 10.1192/bjp.2019.4. PMID: 30767826. [↑](#footnote-ref-26)
27. Guterman NB, Bellamy JL, Banman A, Harty JS, Jaccard J, Mirque-Morales S. Engaging fathers to strengthen the impact of early home visitation on physical child abuse risk: Findings from the dads matter-HV randomized controlled trial. Child Abuse Negl. 2023 Sep;143:106315. doi: 10.1016/j.chiabu.2023.106315. Epub 2023 Jul 5. PMID: 37419071. [↑](#footnote-ref-27)
28. Huang L, Shen Q, Fang Q, Zheng X. Effects of Internet-Based Support Program on Parenting Outcomes for Primiparous Women: A Pilot Study. Int J Environ Res Public Health. 2021 Apr 21;18(9):4402. doi: 10.3390/ijerph18094402. PMID: 33919112; PMCID: PMC8122326. [↑](#footnote-ref-28)
29. Johnston BD, Huebner CE, Anderson ML, Tyll LT, Thompson RS. Healthy steps in an integrated delivery system: child and parent outcomes at 30 months. Arch Pediatr Adolesc Med. 2006 Aug;160(8):793-800. doi: 10.1001/archpedi.160.8.793. PMID: 16894077. [↑](#footnote-ref-29)
30. Kamalifard M, Yavarikia P, Babapour Kheiroddin J, Salehi Pourmehr H, Iraji Iranagh R. The effect of peers support on postpartum depression: a single-blind randomized clinical trial. J Caring Sci. 2013 Aug 28;2(3):237-44. doi: 10.5681/jcs.2013.029. PMID: 25276732; PMCID: PMC4134152. [↑](#footnote-ref-30)
31. Kenyon S, Jolly K, Hemming K, Hope L, Blissett J, Dann SA, Lilford R, MacArthur C. Lay support for pregnant women with social risk: a randomised controlled trial. BMJ Open. 2016 Mar 2;6(3):e009203. doi: 10.1136/bmjopen-2015-009203. PMID: 26936901; PMCID: PMC4785315. [↑](#footnote-ref-31)
32. Kersten-Alvarez LE, Hosman CM, Riksen-Walraven JM, Van Doesum KT, Hoefnagels C. Long-term effects of a home-visiting intervention for depressed mothers and their infants. J Child Psychol Psychiatry. 2010 Oct;51(10):1160-70. doi: 10.1111/j.1469-7610.2010.02268.x. Epub 2010 Aug 12. PMID: 20707826. [↑](#footnote-ref-32)
33. Kiely M, El-Mohandes AAE, El-Khorazaty MN, Gantz MG. An integrated intervention to reduce intimate partner violence in pregnancy: a randomized controlled trial. Obstet Gynecol. 2010 Feb;115(2 Pt 1):273-283. doi: 10.1097/AOG.0b013e3181cbd482. Erratum in: Obstet Gynecol. 2011 May;117(5):1232. Blake, Susan M [added]. PMID: 20093899; PMCID: PMC2917915. [↑](#footnote-ref-33)
34. Leng LL, Yin XC, Chan CLW, Ng SM. Antenatal mobile-delivered mindfulness-based intervention to reduce perinatal depression risk and improve obstetric and neonatal outcomes: A randomized controlled trial. J Affect Disord. 2023 Aug 15;335:216-227. doi: 10.1016/j.jad.2023.04.133. Epub 2023 May 6. PMID: 37156275. [↑](#footnote-ref-34)
35. Maimburg RD, Vaeth M. Postpartum depression among first-time mothers - results from a parallel randomised trial. Sex Reprod Healthc. 2015 Jun;6(2):95-100. doi: 10.1016/j.srhc.2015.01.003. Epub 2015 Jan 15. PMID: 25998877. [↑](#footnote-ref-35)
36. Makrides M, Gibson RA, McPhee AJ, Yelland L, Quinlivan J, Ryan P; DOMInO Investigative Team. Effect of DHA supplementation during pregnancy on maternal depression and neurodevelopment of young children: a randomized controlled trial. JAMA. 2010 Oct 20;304(15):1675-83. doi: 10.1001/jama.2010.1507. PMID: 20959577. [↑](#footnote-ref-36)
37. Maselko J, Sikander S, Bhalotra S, Bangash O, Ganga N, Mukherjee S, Egger H, Franz L, Bibi A, Liaqat R, Kanwal M, Abbasi T, Noor M, Ameen N, Rahman A. Effect of an early perinatal depression intervention on long-term child development outcomes: follow-up of the Thinking Healthy Programme randomised controlled trial. Lancet Psychiatry. 2015 Jul;2(7):609-17. doi: 10.1016/S2215-0366(15)00109-1. Epub 2015 Jun 2. PMID: 26303558. [↑](#footnote-ref-37)
38. McConnell MA, Rokicki S, Ayers S, Allouch F, Perreault N, Gourevitch RA, Martin MW, Zhou RA, Zera C, Hacker MR, Chien A, Bates MA, Baicker K. Effect of an Intensive Nurse Home Visiting Program on Adverse Birth Outcomes in a Medicaid-Eligible Population: A Randomized Clinical Trial. JAMA. 2022 Jul 5;328(1):27-37. doi: 10.1001/jama.2022.9703. Erratum in: JAMA. 2023 Feb 28;329(8):687. PMID: 35788794; PMCID: PMC9257581. [↑](#footnote-ref-38)
39. McFarlane E, Burrell L, Crowne S, Cluxton-Keller F, Fuddy L, Leaf PJ, Duggan A. Maternal relationship security as a moderator of home visiting impacts on maternal psychosocial functioning. Prev Sci. 2013 Feb;14(1):25-39. doi: 10.1007/s11121-012-0297-y. PMID: 23104075; PMCID: PMC3789587. [↑](#footnote-ref-39)
40. Milgrom J, Holt C, Holt CJ, Ross J, Ericksen J, Gemmill AW. Feasibility study and pilot randomised trial of an antenatal depression treatment with infant follow-up. Arch Womens Ment Health. 2015 Oct;18(5):717-30. doi: 10.1007/s00737-015-0512-5. Epub 2015 Feb 24. PMID: 25709044. [↑](#footnote-ref-40)
41. Mohd Shukri NH, Wells J, Eaton S, Mukhtar F, Petelin A, Jenko-Pražnikar Z, Fewtrell M. Randomized controlled trial investigating the effects of a breastfeeding relaxation intervention on maternal psychological state, breast milk outcomes, and infant behavior and growth. Am J Clin Nutr. 2019 Jul 1;110(1):121-130. doi: 10.1093/ajcn/nqz033. PMID: 31161202. [↑](#footnote-ref-41)
42. Nicolson S, Carron SP, Paul C. Supporting early infant relationships and reducing maternal distress with the Newborn Behavioral Observations: A randomized controlled effectiveness trial. Infant Ment Health J. 2022 May;43(3):455-473. doi: 10.1002/imhj.21987. Epub 2022 May 9. PMID: 35531961; PMCID: PMC9324818. [↑](#footnote-ref-42)
43. Olds DL, Robinson J, O'Brien R, Luckey DW, Pettitt LM, Henderson CR Jr, Ng RK, Sheff KL, Korfmacher J, Hiatt S, Talmi A. Home visiting by paraprofessionals and by nurses: a randomized, controlled trial. Pediatrics. 2002 Sep;110(3):486-96. doi: 10.1542/peds.110.3.486. PMID: 12205249. [↑](#footnote-ref-43)
44. Olds DL, Robinson J, Pettitt L, Luckey DW, Holmberg J, Ng RK, Isacks K, Sheff K, Henderson CR Jr. Effects of home visits by paraprofessionals and by nurses: age 4 follow-up results of a randomized trial. Pediatrics. 2004 Dec;114(6):1560-8. doi: 10.1542/peds.2004-0961. PMID: 15574615. [↑](#footnote-ref-44)
45. Olds DL, Kitzman H, Hanks C, Cole R, Anson E, Sidora-Arcoleo K, Luckey DW, Henderson CR Jr, Holmberg J, Tutt RA, Stevenson AJ, Bondy J. Effects of nurse home visiting on maternal and child functioning: age-9 follow-up of a randomized trial. Pediatrics. 2007 Oct;120(4):e832-45. doi: 10.1542/peds.2006-2111. PMID: 17908740; PMCID: PMC2839449. [↑](#footnote-ref-45)
46. Olds DL, Kitzman HJ, Cole RE, Hanks CA, Arcoleo KJ, Anson EA, Luckey DW, Knudtson MD, Henderson CR Jr, Bondy J, Stevenson AJ. Enduring effects of prenatal and infancy home visiting by nurses on maternal life course and government spending: follow-up of a randomized trial among children at age 12 years. Arch Pediatr Adolesc Med. 2010 May;164(5):419-24. doi: 10.1001/archpediatrics.2010.49. PMID: 20439792; PMCID: PMC3249758. [↑](#footnote-ref-46)
47. Onozawa K, Glover V, Adams D, Modi N, Kumar RC. Infant massage improves mother-infant interaction for mothers with postnatal depression. J Affect Disord. 2001 Mar;63(1-3):201-7. doi: 10.1016/s0165-0327(00)00198-1. PMID: 11246096. [↑](#footnote-ref-47)
48. Rahman A, Malik A, Sikander S, Roberts C, Creed F. Cognitive behaviour therapy-based intervention by community health workers for mothers with depression and their infants in rural Pakistan: a cluster-randomised controlled trial. Lancet. 2008 Sep 13;372(9642):902-9. doi: 10.1016/S0140-6736(08)61400-2. PMID: 18790313; PMCID: PMC2603063. [↑](#footnote-ref-48)
49. Robling M, Lugg-Widger FV, Cannings-John R, Angel L, Channon S, Fitzsimmons D, Hood K, Kenkre J, Moody G, Owen-Jones E, Pockett RD, Sanders J, Segrott J, Slater T. Nurse-led home-visitation programme for first-time mothers in reducing maltreatment and improving child health and development (BB:2-6): longer-term outcomes from a randomised cohort using data linkage. BMJ Open. 2022 Feb 10;12(2):e049960. doi: 10.1136/bmjopen-2021-049960. PMID: 35144944; PMCID: PMC8845181. [↑](#footnote-ref-49)
50. Rotheram-Borus MJ, Arfer KB, Christodoulou J, Comulada WS, Stewart J, Tubert JE, Tomlinson M. The association of maternal alcohol use and paraprofessional home visiting with children's health: A randomized controlled trial. J Consult Clin Psychol. 2019 Jun;87(6):551-562. doi: 10.1037/ccp0000408. PMID: 31120274; PMCID: PMC6775769. [↑](#footnote-ref-50)
51. Tomlinson M, Rotheram-Borus MJ, Harwood J, le Roux IM, O'Connor M, Worthman C. Community health workers can improve child growth of antenatally-depressed, South African mothers: a cluster randomized controlled trial. BMC Psychiatry. 2015 Sep 23;15:225. doi: 10.1186/s12888-015-0606-7. PMID: 26400691; PMCID: PMC4581418. [↑](#footnote-ref-51)
52. Tripathy P, Nair N, Barnett S, Mahapatra R, Borghi J, Rath S, Rath S, Gope R, Mahto D, Sinha R, Lakshminarayana R, Patel V, Pagel C, Prost A, Costello A. Effect of a participatory intervention with women's groups on birth outcomes and maternal depression in Jharkhand and Orissa, India: a cluster-randomised controlled trial. Lancet. 2010 Apr 3;375(9721):1182-92. doi: 10.1016/S0140-6736(09)62042-0. Epub 2010 Mar 6. PMID: 20207411. [↑](#footnote-ref-52)
53. Urizar GG Jr, Muñoz RF. Impact of a prenatal cognitive-behavioral stress management intervention on salivary cortisol levels in low-income mothers and their infants. Psychoneuroendocrinology. 2011 Nov;36(10):1480-94. doi: 10.1016/j.psyneuen.2011.04.002. Epub 2011 Jun 8. PMID: 21641117; PMCID: PMC3423333. [↑](#footnote-ref-53)
54. van Doesum KT, Riksen-Walraven JM, Hosman CM, Hoefnagels C. A randomized controlled trial of a home-visiting intervention aimed at preventing relationship problems in depressed mothers and their infants. Child Dev. 2008 May-Jun;79(3):547-61. doi: 10.1111/j.1467-8624.2008.01142.x. PMID: 18489412. [↑](#footnote-ref-54)
55. Walkup JT, Barlow A, Mullany BC, Pan W, Goklish N, Hasting R, Cowboy B, Fields P, Baker EV, Speakman K, Ginsburg G, Reid R. Randomized controlled trial of a paraprofessional-delivered in-home intervention for young reservation-based American Indian mothers. J Am Acad Child Adolesc Psychiatry. 2009 Jun;48(6):591-601. doi: 10.1097/CHI.0b013e3181a0ab86. PMID: 19454915; PMCID: PMC6432645. [↑](#footnote-ref-55)
56. Werner EA, Gustafsson HC, Lee S, Feng T, Jiang N, Desai P, Monk C. PREPP: postpartum depression prevention through the mother-infant dyad. Arch Womens Ment Health. 2016 Apr;19(2):229-42. doi: 10.1007/s00737-015-0549-5. Epub 2015 Aug 2. PMID: 26231973; PMCID: PMC4738166. [↑](#footnote-ref-56)
57. Zielinski DS, Eckenrode J, Olds DL. Nurse home visitation and the prevention of child maltreatment: impact on the timing of official reports. Dev Psychopathol. 2009 Spring;21(2):441-53. doi: 10.1017/S0954579409000248. PMID: 19338692. [↑](#footnote-ref-57)
