# Supplementary material for: Perinatal interventions to prevent Adverse Childhood Experiences (ACEs): A scoping review
Source: PLoS One. 2024 Oct 24;19(10):e0307441. doi: 10.1371/journal.pone.0307441 (PMC11501017; doi:10.1371/journal.pone.0307441)
Supplement: S3 Appendix — (DOCX) [file pone.0307441.s003.docx]

Supplementary Material - Critical Appraisal.

# Key Bias Risk Analysis

Each study was evaluated in terms of whether there was low, high, or unclear risk of the following domains. The domains included:

1. Selection bias - Random sequence generation
2. Selection bias - Allocation concealment
3. Performance bias – Participant blinding
4. Performance bias - Personnel blinding
5. Detection bias - Outcome blinding
6. Attrition bias - Incomplete outcome data

| **Title, Author, Year,**  **Country** | **Impact of study** | **Location / setting of study** | **Adversity focus** | **Selection bias** | | **Performance bias** | | **5. Detection bias**  **(outcome blinding)** | **6. Attrition bias**  **(small, moderate, large)** | **Overall assessment of risk of potential bias**  **(High risk / Low risk / Unclear)** |
| --- | --- | --- | --- | --- | --- | --- | --- | --- | --- | --- |
|  |  |  |  | **1. Random sequence generation** | **2. Allocation concealment** | **3. Participant blinding** | **4. Personnel blinding** |  |  |  |
| 1.  Armstrong et al  2000,  Promoting secure attachment, maternal mood, and child health in a vulnerable population: a randomized controlled trial.  Aust. | Insign, impact mother | - Child health nurse - Home | - Parental ACE history | Low risk | Good | No | Yes | Yes – evaluators were blind | Small attrition | Low |
|  | Sign. impact on child |  |  |  |  |  |  |  |  |  |
| 2.  Austin et al  2008  Brief antenatal cognitive behaviour therapy group intervention for the prevention of postnatal depression and anxiety: a randomised controlled trial.  Aust | Insign. impact mother | - CBT intervention - Group-based - Clinic setting | - Perinatal depression | Low risk | Unclear | No | Yes |  | Large attrition (50% drop out) | Unclear |
|  | Insign impact child |  |  |  |  |  |  |  |  |  |
| 3.  Barlow et al  2007  Role of home visiting in improving parenting and health in families at risk of abuse and neglect: results of a multicentre randomised controlled trial and economic evaluation  UK | Sign. impact mother  (Mothers were sign, more sensitive) | - Family Partnership - Nurse - Home | - Parental ACE history - Social vulnerability | Low risk | Good | No | No | Yes – researchers doing coding were blind | Small attrition (<10%) | Low |
|  | Insign. impact child |  |  |  |  |  |  |  |  |  |
| 4.  Barlow et al  2013  Effect of a paraprofessional home-visiting intervention on American Indian teen mothers’ and infants’ behavioural risks: a randomized controlled trial  US | Sign Impact on mother  (increased parenting knowledge) | - Family Spirit - Paraprofessional - Home | - Rural - Adolescents - Social vulnerability - Rural (Indian reservation) - Adolescents - Social vulnerability | Low risk | Good | No | No | Yes – evaluators were blind | Moderate (20% refusal to participate) | Low |
|  | Sign. impact on child |  |  |  |  |  |  |  |  |  |
| 5.  Barlow et al  2015  Follow up of study above  Paraprofessional-delivered home-visiting intervention for American Indian teen mothers and children: 3-year outcomes from a randomized controlled trial  US | Sign. impact on mother | [follow-up study #4] |  | Low risk | Good | No | No | Yes – evaluators were blind | Moderate (87% participants retained in the control group for the 36-month assessment vs only 78% in the Family Spirit Intervention group.) | Low |
|  | Sign. impact on child |  |  |  |  |  |  |  |  |  |
| 6.  Barnes et al  2009  The utility of volunteer home-visiting support to prevent maternal depression in the first year of life  UK | Nil impact | - Home visiting by volunteers - Home | - Social vulnerability | Low risk | Unclear | Yes | Yes | Moderate - (Multiple challenges with research engagement and recruitment resulted in protocol adjustments.) | Small | Low |
| 7.  Barnes et al  2017  Randomized controlled trial and economic evaluation of nurse-led group support for young mothers during pregnancy and the first year postpartum versus usual care  UK | Nil impact | - Group Family Nurse Partnership (gFNP) - Clinic | - Adolescents - Social vulnerability - At risk child maltreatment | Low risk | Good | No | Yes | Yes | Moderate (problems with recruitment - only 50% agreed to participate) | Low |
| 8.  Bartu et al  2006  Postnatal home visiting for illicit drug-using mothers and their infants: a randomised controlled trial.  Aust | Nil impact | - Home-visiting group (HVG) - Home | - Perinatal Substance use | Low risk | Good | No | No | No – Home visitors collected data | Small attrition | Unclear |
| 9.  Baumgartner et al  2021  Effect of a lay counsellor-delivered integrated maternal mental health and early childhood development group-based intervention in Northern Ghana: A cluster-randomized controlled trial  Nth Ghana | Nil sign. impact | - Group based - Integrated Mothers and Babies Course and Early Childhood Development (iMBC/ECD) program – - Community | - Rural - Social vulnerability | Low risk | Good | No | No | No | Small attrition - Note low uptake of suggested IPV referrals during intervention. | Unclear |
| 10.  Berry et al  2021  Perinatal depression prevention through the mother-infant dyad: The role of maternal childhood maltreatment  US | Impact on mother | - Effective Postpartum Parenting (PREPP) - Clinic based | - Parental ACE history | Low risk | Unclear | No | No |  | Moderate - Attrition during the intervention phase was 17% | Unclear |
|  | Impact on child |  |  |  |  |  |  |  |  |  |
| 11.  Christadoulou  2019  Home visiting and antenatal depression affect the quality of mother and child interactions in South Africa | No impact | - Home visiting - Peer - Home-based | - Social Vulnerability | Unclear | Unclear | No | Yes |  | Small - Only 2% township women did not participate and only 15% drop out by 3 years - high retention. | Low |
| 12.  Cupples et al  2011  RCT of peer-mentoring for first-time mothers in socially disadvantaged areas (the MOMENTS Study)  UK- Ireland | No impact | - (The MOMENTS Study) – - Peer mentoring - Community | - Social vulnerability | Low risk | Good | No | Yes | Yes | Moderate – (64% enrolment rate. High rates of discontinuation of intervention in the intervention group) | Unclear |
| 13.  DuMont et al  2008  Healthy Families New York (HFNY) randomized trial: effects on early child abuse and neglect  US | Sign. impact on mother | - Healthy Families New York (HFNY) - Para-professionals - Home-based | - Social vulnerability - At risk child maltreatment | Low risk | Good | No | No | Unclear | Moderate – (Text states 50% retained in intervention group at 1 year, and 33% retained at 2 years however study retention was much higher- 90% at 1 year) | Unclear |
|  | Sign. impact on child |  |  |  |  |  |  |  |  |  |
| 14.  Eckenrode et al  2000  Preventing child abuse and neglect with a program of nurse home visitation: the limiting effects of domestic violence  US | Some impact mother | - Healthy Families New York (HFNY) - Nurse - Home-based | Social vulnerability | Unclear – (randomization took place in 1978 and prior publications don't state method) | Unclear | No | No | Yes | Small attrition – (Apparent >80% study retention after initial randomization) | Unclear |
|  | Some impact on child |  |  |  |  |  |  |  |  |  |
| 15.  Enoch et al  2016  A Prospective Cohort Study of Influences on Externalizing Behaviors Across Childhood: Results from a Nurse Home Visiting Randomized Controlled Trial  US | Some impact on mother | - Nurse Home Visiting - Home-based | - Social vulnerability | Unclear | Good | No | No | Mixed – (some data collection staff blinded but others not) | Small attrition | Unclear |
|  | Some impact on child |  |  |  |  |  |  |  |  |  |
| 16.  Feinberg et al  2008  Establishing family foundations: intervention effects on coparenting, parent/infant well-being, and parent-child relations  US | Sign. impact on mother | - Family Foundations (FF) - community | - Prevention | Low risk | Good | Yes | Unclear | Unclear | Moderate | Low |
|  | Sign. impact on child |  |  |  |  |  |  |  |  |  |
| 17.  Feinberg et al  2016  Preventive Effects on Birth Outcomes: Buffering Impact of Maternal Stress, Depression, and Anxiety | Sign. impact on mother | - Family Foundations (FF) - community | - Prevention | Unclear | Unclear | No | Unclear | Unclear | Small attrition | Low |
|  | Sign. impact on child |  |  |  |  |  |  |  |  |  |
| 18.  Field et al  2004  Massage therapy effects on depressed pregnant women  US | Sign. impact on mother | - Massage therapy - Community | - Perinatal depression | Unclear | Unclear | No | Unclear | Unclear | Moderate – 25% attrition | Unclear |
|  | Sign. impact on child |  |  |  |  |  |  |  |  |  |
| 19.  Field  2009  Pregnancy massage reduces prematurity, low birthweight and postpartum depression  US | Sign. impact on mother | - Massage therapy - Community | - Perinatal depression | Unclear | Unclear | No | No | No | High – 30% attrition | Unclear |
|  | Sign. impact on child |  |  |  |  |  |  |  |  |  |
| 20.  Field  2012  Yoga and massage therapy reduce prenatal depression and prematurity.  US | Sign. impact on mother | - Yoga and massage therapy - Community | - Perinatal depression | Unclear | Unclear | No | Unclear | Unclear | Moderate – 25% attrition | Unclear |
|  | Sign. impact on child |  |  |  |  |  |  |  |  |  |
| 21.  Fischer et al  2006  Methadone versus buprenorphine in pregnant addicts: a double-blind, double-dummy comparison study  Vienna | Insign impact mother | - Medication - Clinic | - Opioid dependent | Low risk | Good | Yes | Yes | Yes | Moderate – (Large proportion of women screened did not meet the study's criteria. Of 18 enrolled, 4 withdrew and compliance was low) | Unclear |
|  | Insign. Impact child |  |  |  |  |  |  |  |  |  |
| 22.  Fisher et al  2016  Gender-informed, psychoeducational programme for couples to prevent postnatal common mental disorders among primiparous women: cluster randomised controlled trial  Australia | Some impact mother | - WWWT - Psychoeducation group programme - Nurse - Community | - Prevention | Low risk | Good | No | Yes | Yes | Small attrition - (Good recruitment (67%) and high retention (91%)) | Low |
|  | Insign child |  |  |  |  |  |  |  |  |  |
| 23.  Fonagy et al  2016  Randomized controlled trial of parent-infant psychotherapy for parents with mental health problems and young infants  UK | Sign. impact on mother | - PIP psychotherapy - Therapists - Clinic | - Perinatal MH - Social vulnerability | Low | Good | No | ?Yes | Mixed – (Yes for data coders but no for interviewers) |  | Low |
|  | Insign. child |  |  |  |  |  |  |  |  |  |
| 24.  Fraser et al  2000  Home visiting intervention for vulnerable families with newborns: follow-up results of a randomized controlled trial.  Australia | Impact on mother short term only | - Home visiting - Nurse - Home-based | - Prevention and early intervention | Low risk | Good | No | Yes | Yes | Moderate attrition – (68/90 in intervention group retained to 12 months, 70/91 comparison) | Low |
|  | Impact on child – short term only |  |  |  |  |  |  |  |  |  |
| 25.  Guo et al  2020  Preventing Postpartum Depression with Mindful Self-Compassion Intervention: A Randomized Control Study  China | Sign. impact on mother | - Mindful self- compassion - Internet | - Perinatal depression or anxiety | Low risk | Good | No | Yes – internet-based intervention | Yes | Small attrition – (91% study retention) | Low |
|  | Sign. impact on child |  |  |  |  |  |  |  |  |  |
| 26.  Gureje et al  2019  High- versus low-intensity interventions for perinatal depression delivered by non-specialist primary maternal care providers in Nigeria: cluster randomised controlled trial (the EXPONATE trial)  Nigeria | Sign. impact mother | - MH intervention - Midwives (PMCPs) - Clinic | - Perinatal depression | Low risk | Good | No | No | Yes | Moderate (85% retention at 6 months and 79% at 12 months) | Low |
|  | Some impact child |  |  |  |  |  |  |  |  |  |
| 27.  Gutterman et al.  2023  Engaging fathers to strengthen the impact of early home visitation on physical child abuse risk: Findings from the dads matter-HV (DM-HV) randomized controlled trial | Sign. impact when services initiated postnatally | - Home visiting - Professionals and trained paraprofessionals - Home | - Social vulnerability - Eligible for Home visiting programmes | Unclear – utilised people already enrolled into home visiting programmes | Good | Yes | No | Yes | At 12 mth follow up  Control:  Fathers – 71%  Mothers – 81%  Intervention  Fathers – 84%  Mothers – 87% | Low |
|  | Insign. when services initiated prenatally |  |  |  |  |  |  |  |  |  |
| 28.  Huang  2021  Effects of Internet-Based Support Program on Parenting Outcomes for Primiparous Women: A Pilot Study  China | Sign. impact on mother | - Internet | - Prevention PPD | Unclear | Unclear | No | No | Yes | Small attrition – (Small trial 20 in each arm, 10% attrition) | Low |
|  | Insign. child outcomes |  |  |  |  |  |  |  |  |  |
| 29.  Johnston et al  2006  Healthy steps in an integrated delivery system: child and parent outcomes at 30 months  US | Sign. impact on mother | - Healthy Steps - Nurse, social worker and MH clinician - Home-based | - Prevention - PPD | Unclear | Good | No | Unclear | Unclear | Moderate attrition (80% retention) | Unclear |
|  | Sign. impact on child |  |  |  |  |  |  |  |  |  |
| 30.  Kamalifard et al  2013  The effect of peer support on postpartum depression: a single-blind randomized clinical trial  Iran | Sign. impact on mother | - Peer support - Telephone | - Prevention PPD | Low risk | Good | No | No | Yes | Recruitment-half of those eligible for EPDS screening were not screened | Low |
|  | Sign. impact on child |  |  |  |  |  |  |  |  |  |
| 31.  Kenyon et al  2016  Lay support for pregnant women with social risk: a randomised controlled trial  UK | Sign. impact on mother | - Lay support - Clinic and some home-based | - Social vulnerability | Low risk | Good | No | No | Yes | Small attrition - (25/662 women in the intervention arm withdrew as they did not want to meet with their Pregnancy Outreach Worker) | Low |
|  | Sign. impact on child |  |  |  |  |  |  |  |  |  |
| 32.  Kersten-Alverez et  2010  Long-term effects of a home-visiting intervention for depressed mothers and their infants  Netherlands | Insign impact mother | - MH clinician - Home-based | - Reduce Perinatal depression | Low Risk | Good | Yes | Yes | Yes | Moderate attrition – (The attrition rate from T3 to T4 was 18.3%: 4% of the mothers could not be located and 14% refused participation due to family circumstances or not wanting to be reminded of the difficult past) | Low |
|  | Insign. Impact child |  |  |  |  |  |  |  |  |  |
| 33.  Kiely et al  2010  An integrated intervention to reduce intimate partner violence in pregnancy: a randomized controlled trial  US | Sign. impact on mother | - Psycho-behavioural intervention - Clinic | - IPV | Low risk | Good | No | No | Yes | Small attrition | Low |
|  | Sign. impact on child |  |  |  |  |  |  |  |  |  |
| 34.  Leng et al.  2023  Antenatal mobile-delivered mindfulness-based intervention to reduce perinatal depression risk and improve obstetric and neonatal outcomes: A randomized controlled trial. | Sign. Impact on mother | - Psychotherapy - APP based | - Elevated levels of stress | Low risk | Good | No | No | Yes | Small attrition | Low |
|  | Sign. Impact on child |  |  |  |  |  |  |  |  |  |
| 35.  Maimburg, R. D. and M. Vaeth  2015  Postpartum depression among first-time mothers - results from a parallel randomised trial  Denmark | Insign impact mother | - Structured antenatal education programme - Group community class | - Prevention PPD | Low risk | Good | No | Yes | Unclear | Small attrition | Low |
|  | Insign. Impact child |  |  |  |  |  |  |  |  |  |
| 36.  Makrides, M., et al.  2010  Effect of DHA supplementation during pregnancy on maternal depression and neurodevelopment of young children: A randomized controlled trial.  Australia | Insign impact mother | - DHS supplements - Clinic | - Prevention PPD | Low risk | Good | Yes | No | Yes | Unclear  (Numbers of dropouts & reasons given, but no comparison between those who remained and dropouts) | Low |
|  | Insign. Impact child |  |  |  |  |  |  |  |  |  |
| 37.  Maselko, J, et al  2015  Effect of an early perinatal depression intervention on long-term child development outcomes: follow-up of the Thinking Healthy Programme randomised controlled trial.  Pakistan | Insign impact mother | [Follow up study #45] |  | Unclear | Unclear | Yes – (Likely blinded, but possible women could know if in intervention) | Yes – (all fieldworkers were unaware of women's original random assignment) | Yes |  | Low  (strong design generally but recruitment for non-depressed arm was weaker) |
|  | Insign. Impact child |  |  |  |  |  |  |  |  |  |
| 38.  McConnell  2022  US  Effect of an Intensive Nurse Home Visiting Program on Adverse Birth Outcomes in a Medicaid-Eligible Population: A Randomized Clinical Trial | Insign. Mother or child on 1 primary outcome (2 remaining primary outcomes not yet reported).  Some secondary outcomes reported | - Nurses - Home and some telehealth | - Low income | Low risk | Low risk | Yes | Yes – intervenors were not involved in allocation | Yes | Moderate  6516 of the 12589 refused participation (52%).  Unclear if this led to attrition bias | Low |
| 39.  McFarlane, E., et al.  2013  Maternal relationship security as a moderator of home visiting impacts on maternal psychosocial functioning  Hawaii, US | Insign impact mother | - Para-professional - Home-visiting | - Social vulnerability - Assessed at risk of Child Maltreatment | Low risk | Low risk | No | No | Yes | Moderate  Dropouts not well explained | Low |
|  | Insign. Impact child |  |  |  |  |  |  |  |  |  |
| 40.  Milgrom J, et al.  2015  Feasibility study and pilot randomised trial of an antenatal depression treatment with infant follow-up  Australia | Sign impact mother | - CBT couples - Psychologists - Clinic-based | - Reduce PPD | Low risk | Good | No | No | No | Small attrition – programme showed high adherence and acceptability | Low |
|  | Sign. Impact child |  |  |  |  |  |  |  |  |  |
| 41.  Mohd Shukri NH, et al.  2019  Randomized controlled trial investigating the effects of a breastfeeding relaxation intervention on maternal psychological state, breast milk outcomes, and infant behavior and growth.  Malaysia | Some early impact | - Audio-relaxation - Home-based | - Social vulnerability | Low risk | Good | No | No | No | Small attrition – little detail about reason for dropouts | Low  - Low intensity intervention. Outcomes measured not that clinically meaningful |
|  | Insign. Impact later |  |  |  |  |  |  |  |  |  |
| 42.  Nicholson  2022  Supporting early infant relationships and reducing maternal distress with the Newborn Behavioral Observations: A randomized controlled effectiveness trial | Some impact mother.  Some impact on relationship with infant | - Midwife or MCH Nurse - Home-based | - Risk of depression, anxiety and psychosocial factors | Low risk | Good | No | Yes | Yes | Of 295, 254 agreed to participate (86.1% attrition)  Reason for refusal well documented.  Of the 254 screened, 111 (43.9%) screened positive and formed the at-risk subpopulation. | Low |
| 43.  Olds DL, et al.  2002  Home Visiting by Paraprofessionals and by Nurses: A Randomized, Controlled Trial.  US | Both interventions had positive impact mother and child | - Para-professionals and nurses - Home-based | - Social vulnerability | Low risk | Good | No | No | Yes | Unclear – (Don’t give numbers of dropouts or reason, but does note that " the risk profiles of nurse-visited women who dropped out indicate that  they were at lower risk than control group dropouts.  (biasing the study against the nurses)”) | Low |
|  | Small impact para-professionals |  |  |  |  |  |  |  |  |  |
|  | Sign. Impact nurses |  |  |  |  |  |  |  |  |  |
| 44.  Olds, D. L., et al.  2004  Effects of home visits by paraprofessionals and by nurses: Age 4 follow-up results of a randomized trial.  US | Both interventions had positive impact mother and child | - Para-professionals and nurses - Home-based - [Follow up study #40] | - Social vulnerability | Low risk | Good | No | No | Yes | Unclear – (high rate of fu, but don’t explain all drops outs or compare the dropouts from rest) | Low |
|  | Sign. Impact para-prof |  |  |  |  |  |  |  |  |  |
|  | Sign. Impact nurses |  |  |  |  |  |  |  |  |  |
| 45.  Olds, D. L., et al  2007  Effects of nurse home visiting on maternal and child functioning: Age-9 follow-up of a randomized trial.  US | Sign. impact mother | - Nurses - Home-based | - Social vulnerability | Low risk | Good | No | No | Yes |  | Low |
|  | Sign. Impact child |  |  |  |  |  |  |  |  |  |
| 46.  Olds, D. L., et al.  2010  Enduring effects of prenatal and infancy home visiting by nurses on maternal life course and government spending: Follow-up of a randomized trial among children at age 12 years.  US | Sign. impact mother | - Nurses - Home-based - [Follow up study #42] | - Social vulnerability | Low risk | Good | No | No | Yes | Unclear – (don't provide reasons or comparisons of those dropped out from the rest of the sample) | Low |
|  | Insign. Impact child in longer term |  |  |  |  |  |  |  |  |  |
| 47.  Onozawa, K. et al.  2001  Infant massage improves mother-infant interaction for mothers with postnatal depression.  London, UK | Sign. Impact mother | - Massage therapists - Clinic based | - Reduce PPD | Unclear | Unclear | No | No | Yes | don’t explain dropouts, but do a good job of comparing dropouts with rest of sample & no sig difference | Unclear |
|  | Sign. impact child |  |  |  |  |  |  |  |  |  |
| 48.  Rahman, A., et al.  2008  [linked to #36]  Cognitive behaviour therapy-based intervention by community health workers for mothers with depression and their infants in rural Pakistan: A cluster-randomised controlled trial  Pakistan | Sign. Impact mother | - CBT by para-professionals - Home-based | - Reduce PPD - Social vulnerability | Low risk -  (Randomized at union council level. 40 union councils (20 Rx, 20 cntrl)) | Good | Yes | Yes | Yes | Unclear - give dropouts explanations, but no comparison of dropouts vs rest of sample | Low |
|  | Sign. Impact child |  |  |  |  |  |  |  |  |  |
| 49.  Robling et al  2022  Nurse-led home-visitation programme for first-time mothers in reducing maltreatment and improving child health and development (BB:2-6): longer-term outcomes from a randomised cohort using data linkage. | Insign. Impact mother | - Nurses - Home-based | - Young - Social vulnerability | Low risk | Good | Yes | No | Yes | Unclear – not sure what % had complete data set | Low |
|  | Small impact school readiness and attainment at KS1 |  |  |  |  |  |  |  |  |  |
| 50.  Rotheram-Borus, M. J., et al.  2019  The association of maternal alcohol use and paraprofessional home visiting with children’s health: A randomized controlled trial  South Africa | Sign. Impact mother | - Para-professionals - Home-based | - Social vulnerability | Low risk – (Randomized at neighbourhood level - Not fully transparent. Reduced bias by matching on relevant covariates with buffer zones to prevent cross contamination) | Unclear | No | Yes | Yes | Unclear – (show # of deaths as dropouts, but didn't explain other drop outs/non-response or compare dropouts/noresponse to rest of sample) | Unclear |
|  | Some impact child |  |  |  |  |  |  |  |  |  |
| 51.  Tomlinson, M., et al.  2015  Community health workers can improve child growth of antenatally-depressed, South African mothers: a cluster randomized controlled trial  South Africa | Insign. impact Mother | - Para-professionals - Home-based | - Perinatal depression - HIV - Social vulnerability | Unclear – (randomized matched pairs of neighbourhoods (density, public utilities - water, electricity, toilets)) | Unclear | Yes | Yes | Yes | Small attrition – (sample retention high & no sig difference between those retained & dropped off) | Low |
|  | Sign. impact child |  |  |  |  |  |  |  |  |  |
| 52.  Tripathy, P., et al.  2010  Effect of a participatory intervention with women's groups on birth outcomes and maternal depression in Jharkhand and Orissa, India: A cluster-randomised controlled trial  India | Sign. impact mother  (those with mod. depression) | - Volunteers - Community | - Social vulnerability - Rural low-income setting | Low risk | Good | No | No | No | Unclear – (explained but don’t compare dropouts to rest of sample) | Low  – (Important that community partnered/led. Meaningful child outcome) |
|  | Sign. Impact child |  |  |  |  |  |  |  |  |  |
| 53.  Urizar, G. G., Jr.  2011  Impact of a prenatal cognitive-behavioral stress management intervention on salivary cortisol levels in low-income mothers and their infants  US | Sign. Impact mother | - Psychologists - Group-clinic based | - Perinatal Depression, anxiety or stress - Social vulnerability - High risk child maltreatment | Unclear | Unclear | No | Unclear | Unclear | Small attrition – (high adherence, gave reasons and no sig difference between drop out & rest of sample) | Unclear |
|  | Sign. Impact child |  |  |  |  |  |  |  |  |  |
| 54.  Van Doesum KT et al.  2008  A randomized controlled trial of a home-visiting intervention aimed at preventing relationship problems in depressed mothers and their infants.  Netherlands | Sign. Impact mother | - Prevention specialists - Home-based | - Perinatal depression | Low risk | Unclear | No |  | Yes | no sig difference in dropout & rest of sample | Low |
|  | Sign. Impact child |  |  |  |  |  |  |  |  |  |
| 55.  Walkup, J. T., et al  2009  Randomized controlled trial of a paraprofessional-delivered in-home intervention for young reservation-based American Indian mothers.  US | Sign. Impact mother | - Para-professionals - Home-based | - Adolescents - Social vulnerability - Rural setting | Low risk | Good | No | No | No | Moderate - (attrition higher among Rx vs control @ 2mo & 6mo, similar @ 12mo, but drop out not sig diff than rest of sample) | Low |
|  | Sign. Impact child |  |  |  |  |  |  |  |  |  |
| 56.  Werner, E. A., et al.  2016  PREPP: Postpartum depression prevention through the mother–infant dyad  US | Sign. impact mother | - Psychologist - Home-based | - Prevention PPD for mothers at risk | Low risk | High risk | No | No | No | Moderate attrition, provided #s, reasons & compared dropouts to rest of sample | Unclear |
|  | Sign. Impact child |  |  |  |  |  |  |  |  |  |
| 57.  Zielinski, D. S., et al.  2009  Nurse home visitation and the prevention of child maltreatment: impact on the timing of official reports.  US | Insign mother | - Nurses - Home-based | - Social vulnerability - +/-Adolescent | Unclear | Unclear | No | No | Yes | Unclear – (dropouts explained. Assessments were w 81% of those who were originally randomized. Doesn't compare dropouts with rest of sample) | Unclear |
|  | Sign. Impact child |  |  |  |  |  |  |  |  |  |
