# Supplementary material for: Perinatal interventions to prevent Adverse Childhood Experiences (ACEs): A scoping review
Source: PLoS One. 2024 Oct 24;19(10):e0307441. doi: 10.1371/journal.pone.0307441 (PMC11501017; doi:10.1371/journal.pone.0307441)
Supplement: S4 Appendix — (DOCX) [file pone.0307441.s004.docx]

**Appendix 2: Life Course Intervention Research (LCIR) Characteristics**

| **Characteristics** | **Description (Adapted from Russ et al., 2022)** | **Questions** | **The operational definition applied for this study** |
| --- | --- | --- | --- |
| 1. ***Developmentally focused*** | The intervention is tailored to a developmental stage and carefully considers the developmental processes underway during that life stage, with the aim of moving those processes towards health. | How does this study incorporate health development concepts? How might this intervention affect future health development? Are the intervention measures developmentally appropriate? | Intervention designed for expectant parents and infants and initiated in the prenatal period. Intervention addresses topics relevant for these developmental stages. |
| 1. ***Strategically timed*** | Targeted to a critical or sensitive period of development, or a transition or turning point, to intervene with maximum efficacy and impact. Timing is multidimensional, including duration and frequency of intervention, as well as stage of the life course. | Does this intervention target a sensitive time period or transition point? Does it consider/measure how the timing of the intervention affects short- and long-term outcomes? | Intervention targeted to a critical or sensitive period i.e. perinatal period. |
| 1. ***Longitudinally focused*** | Intervention aims to change long-term health trajectories, not just short-term outcomes.  Interventions that improve health reserves and resilience in early life may contribute to disease prevention later in life. | Does the intervention/study consider and measure long-term health impacts and trajectories? Does it consider and measure proximal changes that are precursors to later health gains? | Study reported follow up of 6 months or longer. |
| 1. ***Multi-level / Holistic*** | Intervention is designed to improve >1 aspect of the ecosystem in which children are born, live, learn and grow.  Intervention considers social and cultural contexts, family and community. | What levels of the ecosystem does this intervention target? How does it consider health in a holistic framework? Does the intervention target factors in more than one level that are relevant for outcomes of interest? | Intervention targeted at least mother and infant/ child dyad. Also examined whether intervention targeted wider family, friends and community connections and factors. |
| 1. ***Strengths based*** | Intervention builds on child, youth, family, and community strengths to build health reserves and to create adaptations to circumvent challenges. | What child, youth, family, and community strengths does this intervention consider and build upon? Are there other potential strengths that have not been considered? How might they be incorporated? | Study reported some evaluation of child, youth, family and/or community strengths and tailored the intervention in some way to build on or incorporate these strengths. |
| 1. ***Health optimization focused*** | Intervention aims to optimize health trajectories rather than simply preventing or treating specific health problems.  Intervention addresses flourishing across physical, socioemotional, mental, cognitive and spiritual domains. | How does this intervention contribute to an overall improved health trajectory? Is it focused on something broader that one specific health issue? Are more holistic outcomes considered? | Study addresses positive optimization of health of mother and/or infant rather than a sole focus on illness prevention. |
| 1. ***Health equity focused*** | Intervention supports health equity, recognizing that different circumstances and contexts warrant different intensities of intervention. Intervention is designed to help those with most to gain from its success. | How does this intervention contribute to health equity? Who is likely to benefit most from the intervention? Will there be sufficient sample size from different populations to look at intervention effects by race, gender, SES? Does the study include questions to help answer why there might be differences among groups? Are those differences addressed in the intervention design? Has the intervention been pilot tested with/adapted for different populations? Will it effectively reach the people who need it most? | Intervention specifically targets marginalised or vulnerable groups of people. Also examined whether results reported by race/ethnicity, SES to address potential differences in intervention impact. |
| 1. ***Family-centered*** | Intervention recognizes and supports the unique role of families as incubators of early health development, with potential to build family resilience and buffer children from adverse experiences. | If an individual-level intervention, have you considered how families contribute to the problems and solutions being studied? In what ways might families be incorporated into the intervention design? How might that change outcomes? | Intervention references the role of the family, recognizing their expertise in understanding and impacting their own ecosystem. |
| 1. ***Anti-racist approaches*** | Intervention incorporates ant-racist principles and considers the potential role of racism in impacting a person’s health and development. Incorporates effective, practical responses to racism., including e.g. workforce training, intentionally addressing implicit and explicit bias and all forms of structural racism across systems and structures. | Does the theoretical model consider the potential role of racism and include effective responses? Does the intervention design specifically consider anti-racist research principles? i.e. Does it engage stakeholders from the communities being studied? Does the research team include researchers from different racial, ethnic, and cultural backgrounds (particularly those being studied)? Is there existing work from BIPOC scholars on this topic? | Intervention report includes explicit mention of the potential or actual role of racism or discrimination and any attempt to address it. |
| 1. ***Vertically, Horizontally, and Longitudinally integrated*** | Intervention is integrated with services, programs, and other protective factors, including those outside the medical care sector, at all levels, to create a seamless, forward-leaning, health-optimizing system.  Integration occurs horizontally across sectors, vertically across levels, and longitudinally over time. | What systems impact the target outcomes? How are those systems included in, measured by, and impacted by the intervention design? Could/should other systems be integrated? | Intervention is reported to be linked with other existing health or specialist services, and/ or with external agencies. |
| 1. ***Collaboratively co-designed*** | Intervention is designed by stakeholders (individuals, families, communities) and professionals working together in a collective process within an equal partnership. | At what stages and in what ways are stakeholders (individuals, families, communities) be involved in the design and implementation process? | Stakeholders were reported to be involved in the design phase of the intervention. |
| 1. ***Addresses emerging health development capabilities*** | Intervention is designed to support and enable processes leading to the active development of capacities for positive health, not just management or prevention of disease. | How does this intervention contribute to the acquisition of a positive health development capability? | Intervention supported the acquisition of capabilities or skills that contribute to the development of positive health. |
